# Identification of AMP-activated protein kinase targets by a consensus sequence search of the proteome

**DOI:** 10.1186/s12918-015-0156-0

**Published:** 2015-03-11

**Authors:** Traci L Marin, Brendan Gongol, Marcy Martin, Stephanie J King, Lemar Smith, David A Johnson, Shankar Subramaniam, Shu Chien, John Y-J Shyy

**Affiliations:** Divisions of Biochemistry and Molecular Biology and Biomedical Sciences, University of California, Riverside, CA 92521-0121 USA; Division of Cardiology, Department of Medicine, University of California, San Diego, La Jolla, CA 92093 USA; Division of Physiology, Department of Medicine, University of California, San Diego, La Jolla, CA 92093 USA; Department of Bioengineering and Institute of Engineering in Medicine, University of California, San Diego La Jolla, CA 92093 USA; Department of Cardiopulmonary Sciences and Anatomy, Schools of Allied Health and Medicine, Loma Linda University, Loma Linda, CA 92350 USA

**Keywords:** AMPK, AKT2, ATF2, MMP-2, FOXO3a, NADSYN1, Phosphorylation consensus sequence, Bioinformatics, Proteome, Network prediction

## Abstract

**Background:**

AMP-activated protein kinase (AMPK) is a heterotrimeric serine/threonine protein kinase that is activated by cellular perturbations associated with ATP depletion or stress. While AMPK modulates the activity of a variety of targets containing a specific phosphorylation consensus sequence, the number of AMPK targets and their influence over cellular processes is currently thought to be limited.

**Results:**

We queried the human and the mouse proteomes for proteins containing AMPK phosphorylation consensus sequences. Integration of this database into Gaggle software facilitated the construction of probable AMPK-regulated networks based on known and predicted molecular associations. *In vitro* kinase assays were conducted for preliminary validation of 12 novel AMPK targets across a variety of cellular functional categories, including transcription, translation, cell migration, protein transport, and energy homeostasis. Following initial validation, pathways that include NAD synthetase 1 (NADSYN1) and protein kinase B (AKT2) were hypothesized and experimentally tested to provide a mechanistic basis for AMPK regulation of cell migration and maintenance of cellular NAD^+^ concentrations during catabolic processes.

**Conclusions:**

This study delineates an approach that encompasses both *in silico* procedures and *in vitro* experiments to produce testable hypotheses for AMPK regulation of cellular processes.

**Electronic supplementary material:**

The online version of this article (doi:10.1186/s12918-015-0156-0) contains supplementary material, which is available to authorized users.

## Background

Maintenance of cellular health requires the orchestration of multiple metabolic pathways and signaling cascades whose effects sum to maintain cellular homeostasis. AMP-activated protein kinase (AMPK) has emerged as a major regulator of cellular metabolism through activation of signaling cascades that are protective against stress. AMPK is composed of three subunits (α, β, and γ), and its activation occurs via AMP-dependent and -independent mechanisms leading to phosphorylation of the α subunit at Thr172. While elevation of the AMP: ATP ratio promotes AMP binding to the γ subunit, which permits activation by LKB1, Ca^2+^ influx elicits activation by CaMKKβ independent of the AMP: ATP ratio [1-3]. In addition to Thr172 phosphorylation a variety of posttranslational modifications including phosphorylation at sites other than Thr172 as well as myristolation of the β subunit can control AMPK activation [4].

Once activated, AMPK initiates an array of signaling cascades by phosphorylating proteins with a βϕβXXXS/TXXXϕ consensus sequence (hydrophobic, φ = M, L, I, F, or V; basic, β = R, K, or H, X = any amino acid, S/T = phosphorylation site) [5,6]. AMPK targets often contain slight variants of this consensus sequence, because AMPK can phosphorylate sites flanked by amino acid sequences lacking the basic residues in the −4/-6 positions. Additionally, the −5 hydrophobic residue may be shifted to the −4 position [7]. Understanding how AMPK exerts its protective effects via various signaling pathways requires knowledge of all of AMPK targets, which may be more extensive than currently recognized. To explore this possibility, we mapped its phosphorylation consensus sequence to both human and mouse proteomes. Delineation of AMPK networks involved integration of these data into Gaggle software to organize putative targets based on function. Along with knowledge gained from an extensive literature review, networks were constructed and then used to predict pathways linking the putative targets [8]. This approach yielded an extraordinarily large group of putative AMPK targets and suggested several novel pathways. The predicted pathways from this approach have the potential to redefine the role of AMPK as a survival master switch responding to stress imposed on the cell.

## Results and discussion

### Bioinformatics and systems biology approach

**Figure 1:**
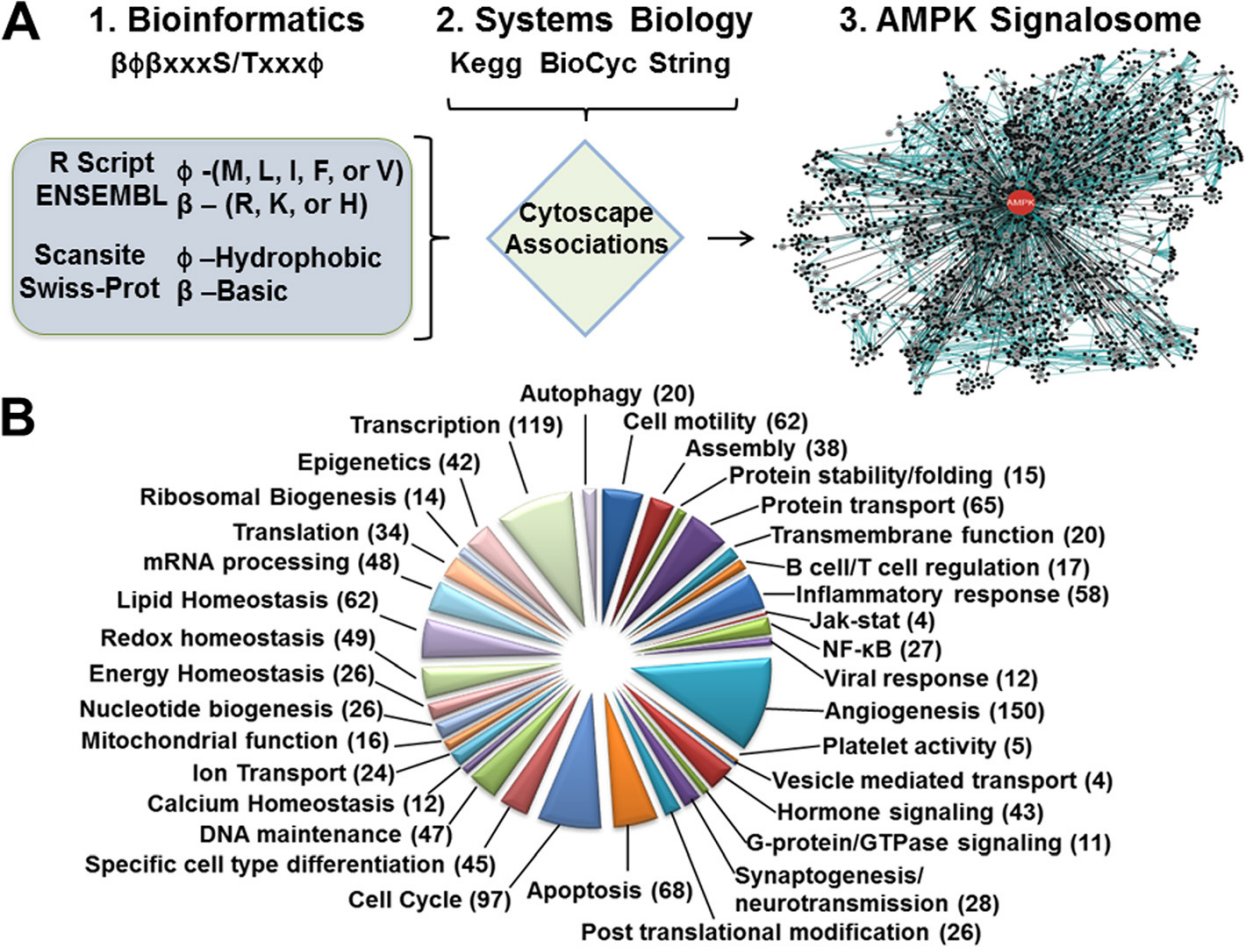
**Bioinformatics screening of putative AMPK substrates. (A)** The algorithm used in the identification of proteins containing an AMPK phosphorylation consensus sequence and their integration into functional categories. **(B)** Pie chart illustrating the representation AMPK consensus phosphorylation sequence containing proteins in various functional categories.

Initially, we performed an *in silico* search of the AMPK phosphorylation consensus sequence to the human and mouse proteomes. The most stringent AMPK consensus phosphorylation sequence (i.e., βϕβXXXS/TXXXϕ [5,6]) was queried to the ENSEMBL proteome, which yielded 866 human and 811 mouse putative AMPK targets. Searching with a less stringent consensus phosphorylation sequence in the SWISS-PROT proteome, in which β and ϕ were unspecified, yielded 4505 human and 3158 mouse putative AMPK targets from over 17,000 peptides. Some putative targets have multiple potential phosphorylation sites. To delineate cellular functions regulated by AMPK via these targets, the resulting databases were imported into Gaggle software, along with putative target annotations compiled from an extensive literature review, and then displayed graphically with Cytoscape software (Figure 1A). This bioinformatics compilation of proteins containing the AMPK phosphorylation consensus sequence represents a novel database for putative AMPK targets and suggests the involvement of AMPK in cellular functions spanning both top-down (receptor response to stimulus) and bottom-up perspectives (transcriptional regulation for cellular functions) (Figure 1B). A representative list of potential AMPK targets is found in Additional file 1: Table S1.

### AMPK phosphorylation of NADSYN1 and transactivation through FOXO3a

**Figure 2:**
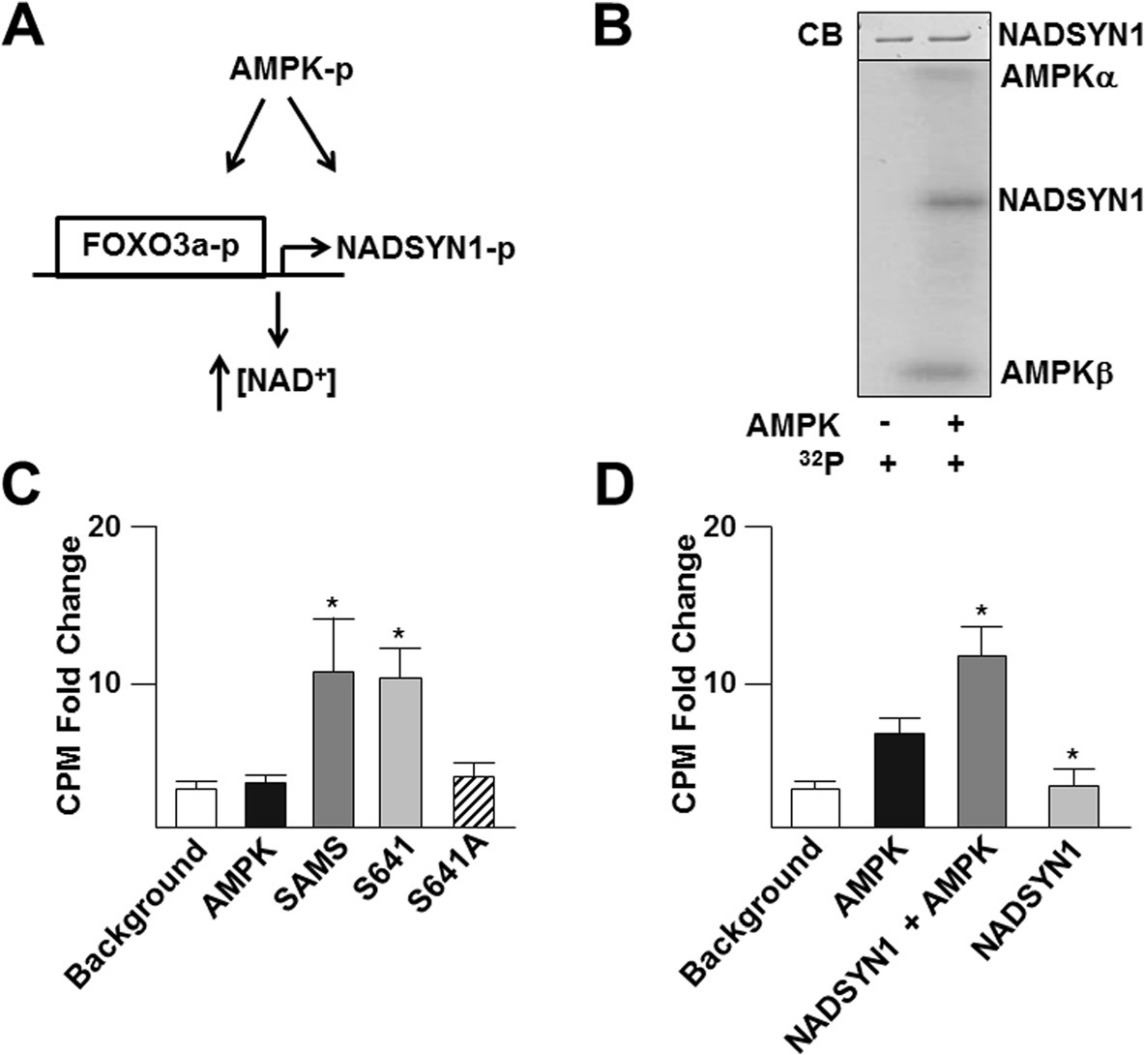
**AMPK phosphorylates NADSYN1 at Serine 641. (A)** Schematic of AMPK phosphorylation and transactivation of NADSYN1. **(B)** Bottom panel represents *in vitro* kinase assay using (γ-^32^P) ATP, full-length recombinant NADSYN1 without (left) and with recombinant (right), active AMPK. Top panel represents to Coomassie blue (CB) stain to demonstrate equal substrate protein loading. Scintillation counter per minute (CPM) quantification of kinase assay repeated using NADSYN1 Ser641 and Ser641A peptides **(C)** or full length NADSYN1 **(D)**. Background represents reactions with only (γ-^32^P) ATP without AMPK nor substrate. AMPK represents reactions with AMPK, (γ-^32^P) ATP, but no substrate. SAMS is a peptide containing the AMPK phosphorylation site in acetyl CoA carboxylase, serving as a positive control. Student t-test used to determine *p < 0.5, all experiments were repeated at least 3 times.

**Figure 3:**
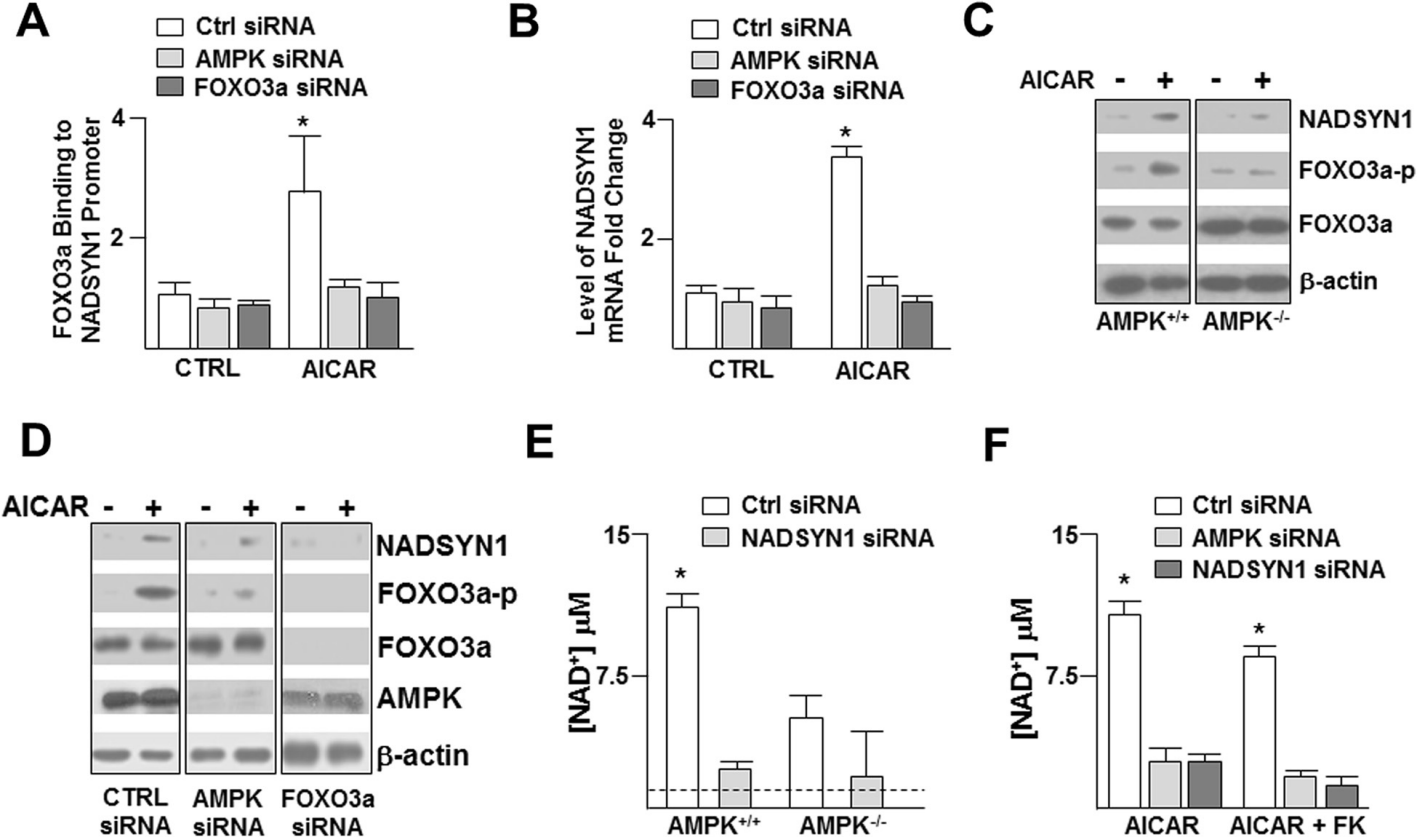
**AMPK activation increases the expression and activity of NADSYN1. (A)** ChIP assay illustrating the level of FOXO3a binding to the NADSYN1 promoter following treatment with AMPK activator, 5-amino-1-β-D-ribofuranosyl-imidazole-4-carboxamide (AICAR, 1 mM), for 8 h. **(B)** qRT-PCR results illustrating the NADSYN1 mRNA abundance following transfection with control, AMPK, or FOXO3a siRNA and then treated with AICAR or left untreated. **(C)** Immunoblot of AMPK^+/+^ and AMPK^−/−^ MEFs transfected with control, AMPK, or FOXO3a siRNA and treated with AICAR or left untreated. **(D)** Immunoblotting of lysates from cells transfected with control, AMPK, or FoxO3a siRNA then treated with or without AICAR. [NAD^**+**^] measured in AMPK^+/+^ and AMPK^−/−^ MEFs (dashed line represents CTRL no treatment) **(E)** or C2C12 cells **(F)** transfected with control, AMPK, or NADSYN1 siRNA and treated with AICAR with or without the NAMPT inhibitor FK688. Data analyzed using Wilcoxon signed-rank test or Mann–Whitney U test. *p < 0.05, all experiments were repeated at least 3 times.

We next focused on an array of cellular processes that appear to be regulated by AMPK for validation and partial pathway delineation. Acting as an energy homeostatic regulator, AMPK activation should increase NADH levels necessary for ATP generation through oxidative phosphorylation and prolonged catabolic flux. Cantó *et al.* reported that AMPK activation increases cellular NAD^+^ levels independent of nicotinamide phosphoribosyltransferase (NAMPT), a key enzyme in NAD^+^ synthesis [9]. Of the pathways involving NAD^+^ biogenesis, glutamine-dependent NAD^+^-synthetase (NADSYN1) also contributes to maintaining cellular NAD^+^ by catalyzing the final step in its biosynthesis. NADSYN1 and NAD^+^ are precursors for many signaling molecules involved in a variety of cellular processes, particularly for redox balance [10]. Furthermore, AMPK can directly phosphorylate the transcription factor, forkhead box O3a (FOXO3a), which likely binds to the NADSYN1 promoter [11]. Given that NADSYN1 contains an AMPK phosphorylation consensus sequence and its promoter has a FOXO3a recognition sequence, we hypothesized that AMPK phosphorylates and activates NADSYN1 as well as promotes NADSYN1 transcription through phosphorylation of FOXO3a (Figure 2A). *In vitro* kinase assays of recombinant NADSYN1 showed that AMPK can phosphorylate NADSYN1 (Figure 2B). Elevated AMPK activity in these assays was evident by AMPK autophosphorylation (Figure 2B). To demonstrate that AMPK can phosphorylate the predicted target site at Ser641, we generated a peptide analogous to the sequence containing Ser641 and its flanking AMPK phosphorylation consensus sequence (KVKRFFS_641_KYSM) and a mutant peptide with Ser to Ala substitution. *In vitro* kinase assay in the presence of AMPK showed increased ^32^P incorporation to the S641 peptide and SAMS peptide, a 13-residue peptide with a sequence around the AMPK target on acetyl CoA carboxylase and the standard positive control for AMPK kinase activity [5] (Figure 2C). The level of ^32^P incorporated into the S641A peptide was much lower, which was comparable to background or reaction without peptide. Similar results were obtained with the use of full length recombinant NADSYN1 (Figure 2D). Next, we investigated whether AMPK regulates NADSYN1 transactivation through phosphorylation of FOXO3a. Chromatin immunoprecipitation (ChIP) assays revealed that AICAR, an AMPK agonist, increased the binding of FOXO3a to the NADSYN1 promoter with a corresponding increase in the level of NADSYN1 mRNA in C2C12 cells (Figures 3A, 3B), which were attenuated if AMPK or FOXO3a were knocked down by siRNA. Additionally, AICAR increased the level of phosphorylated FOXO3a and NADSYN1 in AMPK^+/+^, but not AMPK^−/−^ mouse embryonic fibroblasts (MEFs) (Figure 3C). We also verified AMPK phosphorylation of NADSYN1 by using C2C12 cells, which are a mouse muscle cell line. Likewise, AICAR increased NADSYN1 expression and FOXO3a phosphorylation in C2C12 cells (Figure 3D). This increase was abolished when AMPK or FOXO3a were knocked down. Next, we investigated how NADSYN1 activity is affected by AMPK phosphorylation. As illustrated in Figure 3E, the AMPK^+/+^ MEFs had a higher level of [NAD^+^] compared to AMPK^−/−^ MEFs, which was attenuated when NADSYN1 was knocked down. In addition to NADSYN1, NAMPT is also a source of intracellular NAD^+^. Following transfection with control, AMPK, or NADSYN1 siRNA, C2C12 cells were treated with AICAR in the presence or absence of FK688, a pharmacological inhibitor of NAMPT, and the [NAD^+^]/[NADH] ratio was measured. While AMPK activation by AICAR increased the cellular [NAD^+^], NADSYN1 or AMPK knocked down attenuated this increase (Figure 3F). Taken together, these results suggest that AMPK phosphorylates NADSYN1 to prolong NAD^+^ production, while concurrently increasing NADSYN1 transactivation through phosphorylation of FOXO3a. Because of the involvement of AMPK, FOXO3a, and [NAD^+^] in regulating cellular energy status, this proposed pathway can be used to test hypotheses involving AMPK’s regulated metabolic pathways during energy consuming processes such as cell migration or angiogenesis.

### AMPK activation increases cell migration through the AKT2-ATF2-MMP-2 pathway

**Figure 4:**
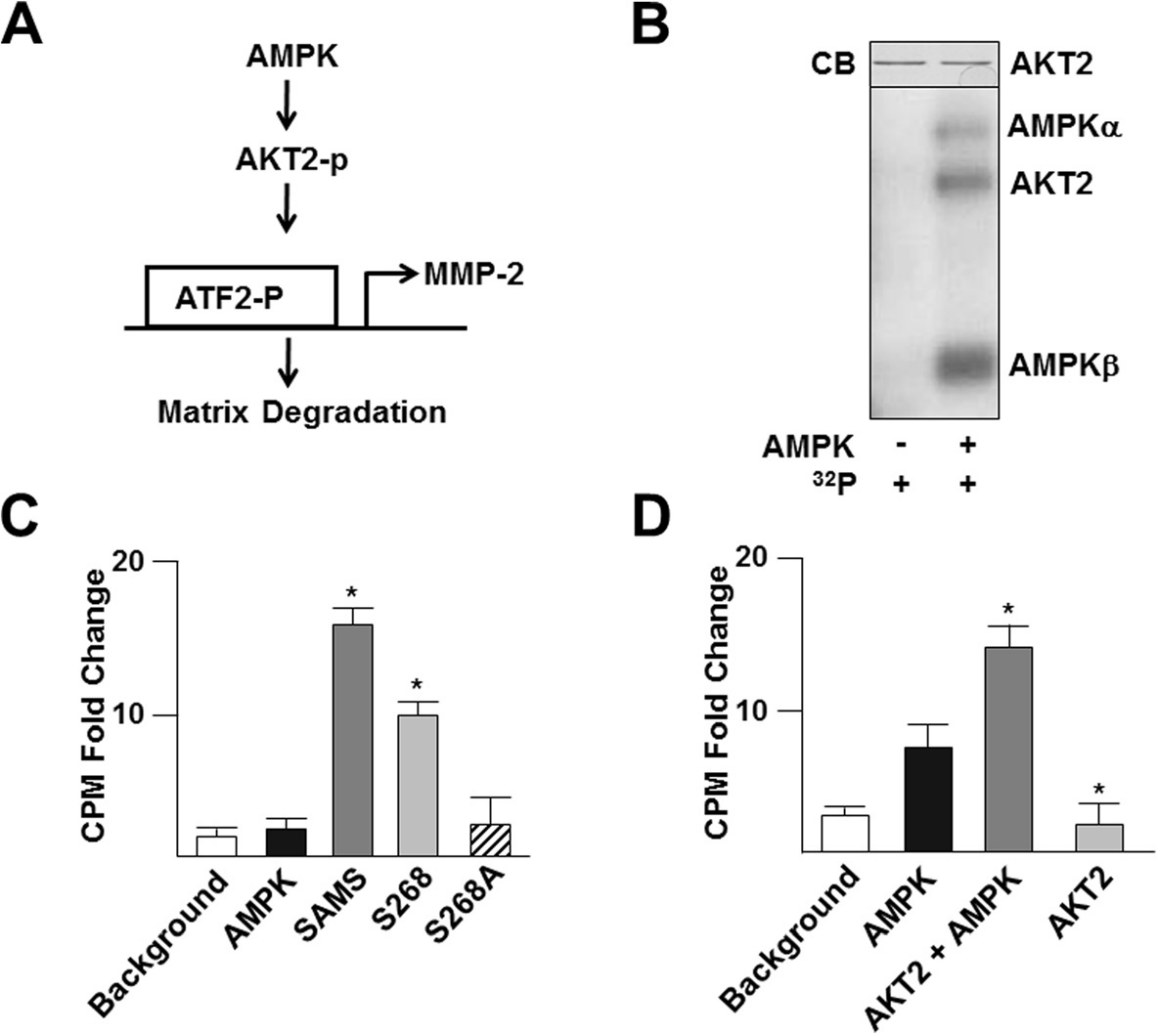
**AMPK phosphorylation of AKT2 at Serine 268. (A)** Schematic of AMPK regulation of EC migration through AKT2 phosphorylation. **(B)** Bottom panel represents *in vitro* kinase assay using (γ-^32^P) ATP and full-length recombinant AKT2 in the presence or absence of AMPK. Top panel represents CB staining for equal loading of recombinant AKT2. (C,D) CPM quantification of kinase assay using AKT2 Ser268 and Ser268A peptides **(C)** or full-length AKT2 **(D)**. The experimental conditions were the same as those in Figure. 2. Student t-test used to determine *p < 0.5, all experiments were repeated at least 3 times.

**Figure 5:**
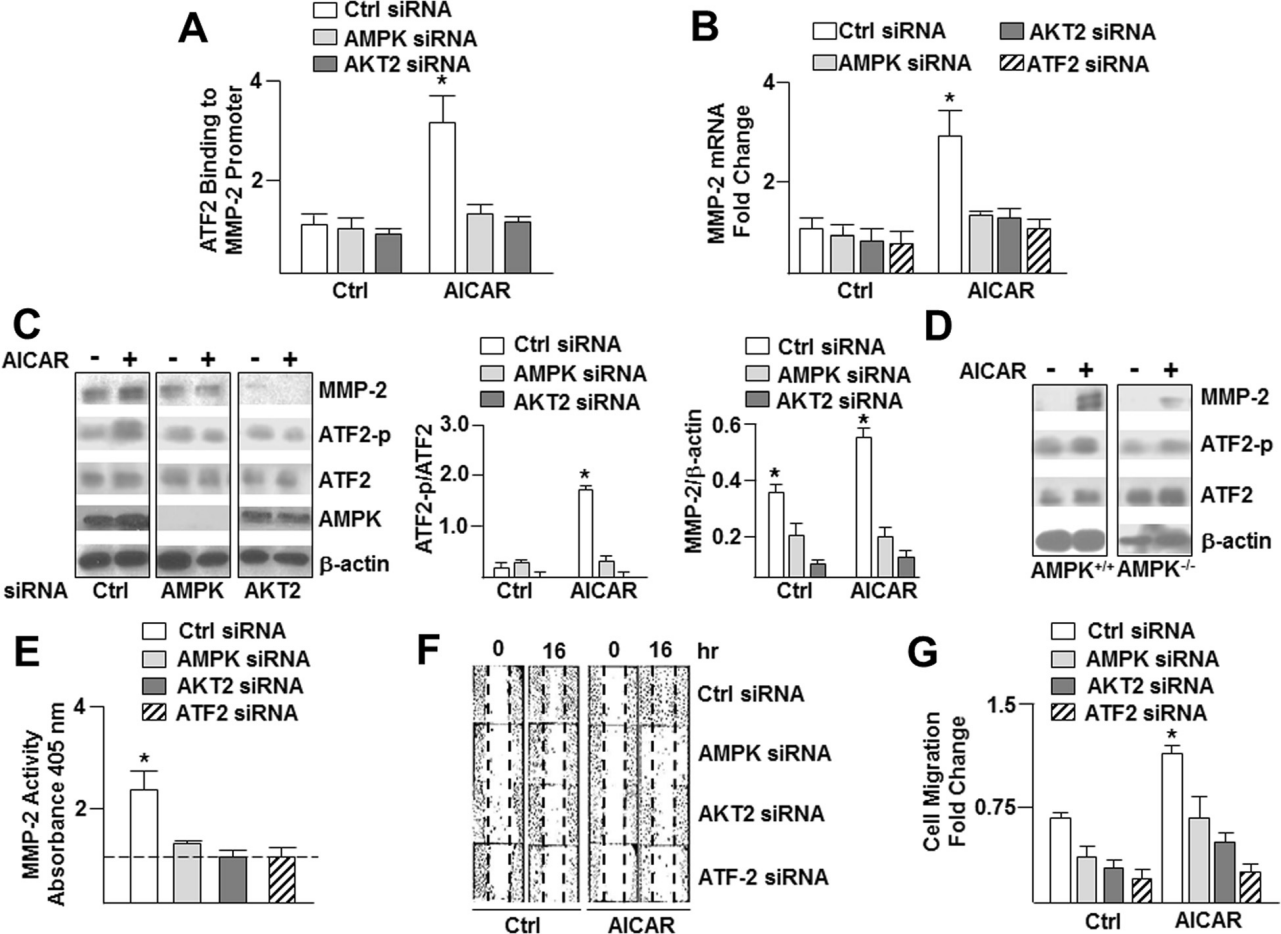
**AMPK activation regulates EC migration through transactivation of MMP-2. (A)** ChIP analysis illustrating the level of ATF2 binding to the MMP-2 promoter in HUVECs transfected with control, AMPK, or AKT2 siRNA, then treated with AICAR (1 mM). Results are expressed as fold change. **(B)** HUVECs were transfected with control, AMPK, AKT2, or ATF2 siRNA and treated with AICAR prior to quantifying MMP-2 mRNA abundance with qRT-PCR. Immunoblot analysis and densitometry of MMP-2 and ATF2 (phosphorylated and total) in **(C)** HUVECs transfected with control, AMPK, or AKT2, siRNA or **(D)** AMPK^+/+^ and AMPK^−/−^ MEFs treated with AICAR or left untreated. **(E)** HUVECs were transfected with control, AMPK, AKT2, or ATF2 siRNA and then treated with AICAR or left untreated for 8 h prior to analyzing the level of MMP-2 activity. **(F)** Scratch assay with confluent ECs transfected with control, AMPK, AKT2, or ATF2 siRNA followed by scratch induction then treated with AICAR or left untreated for 8 hr prior to imaging and quantifying EC migration **(G)**. Data analyzed using Wilcoxon signed-rank test or Mann–Whitney U test. *p < 0.05, all experiments were repeated at least 3 times.

AMPK is involved in a variety of stress responses of vascular endothelial cells (ECs) such as cell migration and angiogenesis, both of which require prolonged metabolic flux to achieve energy homeostasis. For example, AMPK positively regulates EC migration and differentiation facilitating angiogenesis under hypoxia [12,13]. With our phosphorylation consensus sequence database, we assembled a subgroup of putative AMPK targets involved in angiogenesis. Within this subgroup, we chose AKT2 for analysis because AKT2 phosphorylates activating transcription factor 2 (ATF2) [14,15]. In turn, phosphorylated ATF2 transcriptionally regulates matrix metalloproteinase-2 (MMP-2) causing matrix degradation and cell migration [16,17], as summarized in Figure 4A. Given that AKT2 Ser268 is a putative AMPK phosphorylation site, we performed kinase assays and quantified the level of phosphorylation using both the recombinant AKT2 (Figures 4B, 4D) and peptides analogous to a sequence around Ser268 that includes the AMPK phosphorylation consensus sequence (LEYLHS_268_RDVV) or a peptide with an alanine substituted for the serine to support AMPK phosphorylation of AKT2 at Ser268 (Figure 4C). Next, we conducted ChIP assays to determine if AMPK phosphorylation of AKT2 increases ATF2 binding to the MMP-2 promoter. ECs treated with AICAR had an increased level of ATF2 interacting with the MMP-2 promoter but not if AMPK or AKT2 were knocked down (Figure 5A). Furthermore, AICAR treatment increased MMP-2 mRNA levels, which were attenuated if the cells were transfected with AMPK, AKT2, or ATF2 siRNA (Figure 5B). Additionally, MMP-2 protein levels also increased with AICAR treatment in ECs transfected with control RNA, but not in cells transfected with AMPK or AKT2 siRNA (Figure 5C). Immunoblotting further revealed that AICAR increased MMP-2 expression and phosphorylation of ATF2 in AMPK^+/+^ but not in AMPK^−/−^ MEFs (Figure 5D). Next, we tested if AMPK regulates the activity of MMP-2. As illustrated in Figure 5E, AMPK activation by AICAR increased MMP-2 activity but not if the cells were transfected with AMPK, AKT2 or ATF2 siRNA. We then conducted *in vitro* wound-closure assays to elucidate the effect of the AMPK/AKT2/ATF2/MMP-2 signaling cascade on EC migration and model wound healing. Cells treated with AICAR showed enhanced cell migration into the scratched zone, which was attenuated if AMPK, AKT2, or ATF2 were knocked down (Figures 5F, 5G). Taken together, these data suggest that AMPK regulates EC migration through phosphorylation of AKT2, which promotes ATF2 transactivation of MMP-2 during EC migration.

### Identification of novel AMPK substrates and signaling networks for future study

**Figure 6:**
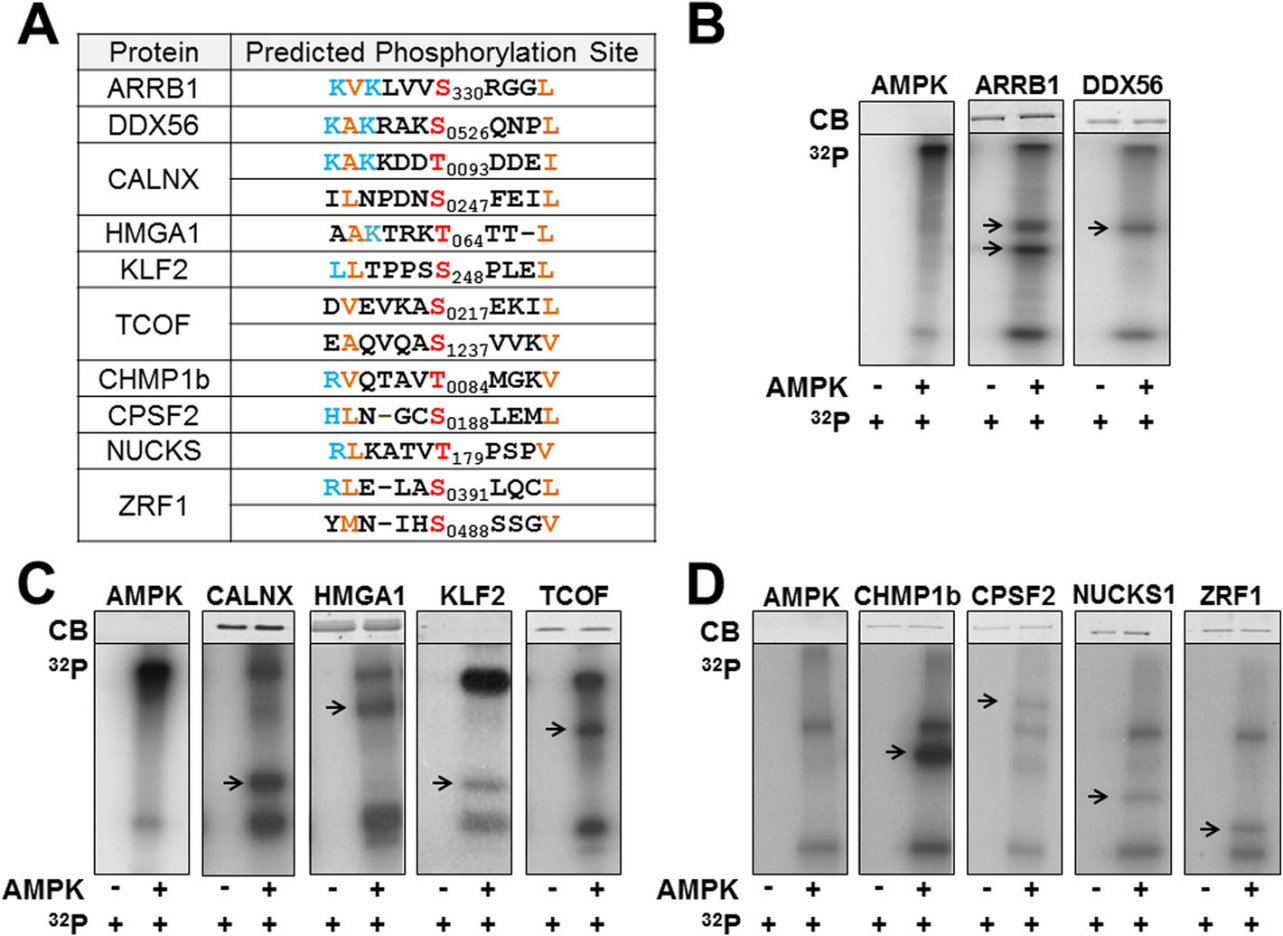
**Kinase assays and predicted phosphorylation sequence of representative AMPK targets. (A)** AMPK phosphorylation consensus sequence. Blue represents basic amino acids, orange represents hydrophobic amino acids, and red represents phosphorylated amino acids. **(B-D)** Coomassie blue staining (CB) and autoradiography of *in vitro* kinase assays without (left) and with activated AMPK (right), (γ-^32^P) ATP, and full-length recombinant substrates. Arrow (→) indicates substrate. Each panel is accompanied by the control kinase assay that contained only recombinant, active AMPK and (γ-^32^P) ATP to identify relative position of autophosphorylated AMPK subunits.

After assembling a list of proteins containing the AMPK phosphorylation consensus sequence, the resulting database was integrated into Gaggle software to gain an understanding of the subcellular systems or functional categories that the identified targets represent. We also utilized this software to determine the number of connections each target has with other proteins. Central node substrates were identified as proteins with a high level interconnectivity within a functional category, which indicates a greater influence a protein has on a phenotypic response. Highly connected nodes were chosen for further validation as AMPK targets using kinase assays. Predicted phosphorylation sequences of the identified proteins chosen are illustrated in Figure 6A. Kinase assays demonstrate AMPK can phosphorylate these full length proteins (Figures 6B-D). Based on these results, we outlined micro networks illustrating the outcome AMPK phosphorylation of each substrate may have. Additional file 2: Figure S1 illustrates graphically how these substrates could be involved in various cellular functions.

### G protein coupling to the seven-transmembrane receptor (GPCRs) activity

Beta Arrestin 1 (ARRB1) regulates many signaling cascades through steric hindrance of GPCRs and serves as a scaffold for several AMPK regulated signaling molecules, including extracellular-signal-regulated kinases 1/2 (ERK1/2) and nuclear factor kappa-light-chain-enhancer of activated B cells (NFκB) [18,19]. Additionally, it participates in transactivation of c-fos and p27 by recruiting histone acetyltransferases, such as p300, which correlates with conditions that activate AMPK [20-22]. Thus, AMPK’s regulation of these signaling pathways could be mediated through ARRB1 phosphorylation (Additional file 2: Figure S1A).

### 5’-terminal oligopyrimidine (TOP) regulation

DDX56, also known as nucleolar helicase of 61 kDa (NOH61), is a putative RNA helicase implicated in a number of cellular processes involving alteration of RNA secondary structure [23]. DDX56 is a constituent of free nucleoplasmic 65S preribosomal particles and seems to be necessary for ribosome synthesis at the level of large (60S) ribosomal subunit assembly [23]. Transcriptional inhibitors, such as actinomycin D, cause complete dissociation of DDX56 from nucleolar components [23]. All vertebrate genes encoding for the ribosomal proteins contain TOP [23]. Interestingly, exercise-activated AMPK is accompanied by decreased translation of TOP containing genes [24]. Although the mechanism by which activated AMPK represses the translation of ribosomal proteins is unclear, it would be independent of ribosomal protein S6 kinase or ribosomal protein S6 (rpS6) phosphorylation, both of which are known to regulate translation [25]. One possibility is that AMPK decreases the translation of TOP genes through phosphorylation of DDX56 and in turn attenuates the functions related to TOP genes in the nucleolus (Additional file 2: Figure S1B).

### Endoplasmic reticulum (ER) stress and calcium homeostasis

Calnexin (CALNX) is a type I integral ER transmembrane chaperone that transiently binds to the newly synthesized glycoproteins to assist in their folding and transport [26]. Because ER stress is hallmarked by elevated levels of unfolded organelle proteins [27], CALNX has been suggested to be a key protective component of the unfolded protein response (UPR)/ER stress response, which activates protein folding/transport chaperones, while decreasing protein synthesis [28]. Through phosphorylation of its cytoplasmic domain, CALNX also regulates sarco/endoplasmic reticulum calcium ATPase (SERCA) 2b, regulating Ca^2+^ signaling and Ca^2+^-sensitive chaperone functions in the ER [29]. This phosphorylation event prevents the inhibition of SERCA and the subsequent Ca^2+^ efflux to cause ER stress [30]. Although AMPK decreases SERCA oxidation, maintaining its activity and intracellular Ca^2+^ homeostasis [31], the mechanism for this is unknown. Our analyses suggest that AMPK regulation of Ca^2+^ and SERCA activity could be through phosphorylation of CALNX Ser-247 and/or Thr-93, functioning as a protective mechanism (Additional file 2: Figure S1C).

### Insulin signaling and glucose transport

High-mobility group AT-hook 1 (HMGA) is a transcriptional regulator that preferentially binds to the minor groove of A/T-rich regions in double-stranded DNA to form transcriptionally active multiprotein-DNA complexes that regulate the expression of target genes such as the insulin receptor (IR) [32]. HMGA1, through AMPK-caveolin-1 (Cav-1) signaling, can also potentiate the recruitment of transcriptional complexes to the solute carrier family 2 and glucose transporter member 3 (SLC2A3/Glut3) promoters [33]. Importantly, Cav-1 expression is necessary for metformin induction of AMP binding and activation of AMPK [34]. These observations suggest that the Cav-1/AMPK/HMGA1 signaling pathway is involved in the transcriptional regulation of genes important for insulin signaling and glucose transport (Additional file 2: Figure S1D).

### Smooth muscle cell (SMC) recruitment and vasculogenesis

Krüppel-like Factor 2 (KLF2) is a transcription factor that regulates diverse biological processes by direct DNA binding or association with transcription co-regulators such as acetyltranferases, cAMP response element binding protein (CREBP), and p300 [35-37]. KLF2 is highly expressed in ECs during embryonic development [38]. *Klf2* genetic knockout results in defective recruitment of pericytes and vascular SMCs, impaired vasculogenesis, and lethality [39]. The mechanism by which KLF2 recruits SMCs is poorly understood. Lysophosphatidic acid (LPA) and its receptor, LPA4, are mediators of SMC recruitment in vasculogenesis. Interestingly, the *LPA4* promoter contains a KLF2 binding site [40]. Given that AMPK activation promotes KLF2 expression [41], AMPK may modulate KLF2 transcriptional activity to increase LPA4 expression (Additional file 2: Figure S1E). Our analysis suggests that AMPK phosphorylation of KLF2 might provide an additional mode of regulation such as enhanced protein stability, altered protein-protein/protein-nucleic acid interactions, or increased transcriptional specificity.

### Ribosomal biogenesis

Treacle, commonly referred to as TCOF, is a nucleolar protein involved in ribosomal gene transcription. TCOF interacts with upstream binding factor (UBF) and promoter selectivity factor SL1 to promote RNA polymerase I activity [42,43]. TCOF deficiency results in poor neural crest formation and proliferation due to neuroepithelial apoptosis as a consequence of decreased expression of 28s ribosomal subunit of rRNA, which ultimately results in the expression of genes important for structural development [44]. Although the role of AMPK in regulating protein synthesis is generally believed to be negative, AMPKα_1_ and α_2_ null embryos are lethal ~10.5 days post-conception because of poor expression of genes important for growth and survival [45]. If AMPK indeed regulates TCOF expression, it may prevent aberrant protein synthesis allowing normal embryonic development (Additional file 2: Figure S1F).

### Epigenetics

Charged multivesicular body protein 1B (CHMP1b) also plays a role in gene regulation through chromatin structural maintenance [46]. CHMP1b associates with nuclease-resistant, condensed chromatin and the polycomb-group (PcG) proteins, which are required for maintenance of gene silencing during development. CHMP1b induction causes cell-cycle arrest and increased S-phase cell number [47]. However, CHMP1b also forms a shell around chromatin that frequently is associated with histone H3 phosphorylation and acetylation regulating transcriptional activity [47,48]. The phosphorylation of CHMP1b by AMPK could play a role in the transition of active and inactive chromatin domains and therefore determination of heritable epigenetic marks (Additional file 2: Figure S1G).

### mRNA processing and translation

Cleavage and polyadenylation specificity factor 2 (CPSF2) is the 100-kDa subunit of the CPSF that recognizes the AAUAAA consensus sequence and interacts with proteins such as the poly (A) polymerase to prompt cleavage of template mRNA and initiation of poly (A) addition in pre-mRNA 3'-end formation [49]. This process inhibits mature mRNA degradation and activates translation [49]. Under stress, AMPK phosphorylation of CPSF2 may increase the association of CPSF2 with the CPSF complex, thus directing CPSF’s recognition of the AAUAAA sequence (Additional file 2: Figure S1H). As a result of this process, AMPK could increase the translation of genes necessary for stress resistance. Alternatively, AMPK could inhibit this process causing the degradation of genes that are involved in excessive energy expenditure.

### Cell cycle and circadian rhythm

Nuclear casein kinase and cyclin-dependent kinase substrate 1 (NUCKS1) is a substrate for casein kinase and cyclin-dependent kinase (CDK) and its phosphorylation is important for cell-cycle regulation [50]. Upon mitosis, DNA-bound NUCKS1 translocates from the nucleus to the cytoplasm [51]. Aberrant overexpression of NUCKS1 is correlated with breast carcinomas [50,51]. Importantly, cancer is highly associated with disrupted cellular circadian rhythm, which serves to regulate positive and negative transcriptional feedback loops. Both casein kinase and CDK are key regulators of cellular circadian rhythm as is AMPK [52,53]. Thus, AMPK could regulate circadian rhythm through phosphorylation of NUCKS1 (Additional file 2: Figure S1I).

### Chromatin structure and protein folding

Zuotin-related factor 1 (ZRF1) executes both cytosolic and nuclear functions. In the nucleus, ZRF1 binds to the DNA sequence: 5’-GTCAAGC-3’ and facilitates histone 2A K119 ubiquitination leading to chromatin remodeling and transcription activation [54]. In the cytoplasm, ZRF1, by stimulation of the ATPase activity of heat shock 70 kDa protein A14 (HSPA14) chaperones, acts as a molecular chaperone to promote protein folding of the nascent polypeptide chain as it exits the ribosome [55,56]. The phosphorylation of ZRF1 by AMPK may result in dissociation of ZRF1 from the ribosome leading to its nuclear accumulation (Additional file 2: Figure S1J). Thus, this pathway putatively regulated by AMPK would participate in epigenetic remodeling of promoters of target genes.

## Conclusion and perspective

AMPK has emerged as a master regulator of metabolism by serving as an energy sensor. However, the multiple modes of AMPK activation, many of which remain elusive, indicate that AMPK’s function may extend beyond a mere energy sensor. Here, our *in silico* database together with biological validations suggests that AMPK is a master regulator of a broader range of cellular processes at more levels than previously recognized. Identifying AMPK targets by phosphorylation consensus sequence mapping is a simplification of the biochemistry that occurs at the substrate-enzyme interface because tertiary structure and binding domain chemistry is important for kinase substrate specificity and accessibility. However, arguably, the constraints of the active site require a minimum signature sequence within the catalytic pocket that mirrors a chemical signature flanking the phosphorylation site for catalysis to occur [57]. Although *in silico* screening of the phosphorylation consensus sequence for candidate substrates of a kinase is a useful tool, the presence of such a phosphorylation consensus sequence in a given protein does not necessitate its phosphorylation in the cell. Further, some predicted AMPK targets offer slight variation to the exact consensus sequence, which presents a limitation of *in silico* analysis. Clearly, additional validation of individual targets and networks is necessary. Several biochemical tools are available and essential for such validation including *in vitro* kinase assays using recombinant protein substrates and phospho-antibodies for cellular and *in vivo* studies. Additional levels of AMPK phosphorylation specificity can be achieved with AMPK α1 and α2 knockout MEF cells and conditional knockout mice following treatment with AMPK agonists/activators such as exercise, caloric restriction, metformin, and AICAR. This database provides investigators with an AMPK-related network development tool that should stimulate experimental validation and offers new insights into AMPK involvement in health and disease.

## Methods

### Bioinformatics approach

Using an R script, the AMPK phosphorylation consensus sequence, βϕβXXXS/TXXXϕ (where hydrophobic, φ = M, L, I, F, or V; basic, β = R, K, or H, X = any amino acid, S/T = phosphorylation site) was mapped to human and mouse proteomes imported from ENSEMBL proteome database [5-7]. To find the proteins containing an AMPK phosphorylation consensus sequence, each peptide sequence obtained from the R script mapping was individually pasted into the BLAST algorithm provided by NCBI (http://www.ncbi.nlm.nih.gov/sutils/blink.cgi?mode=query). Using the less stringent consensus phosphorylation sequence, in which φ and β were not specified to be a specific amino acid, a Scansite search was also performed to compare with the ENSEMBL search to the SWISS-PROT protein database [5-7]. The generated databases were integrated into Gaggle software to predict and display pathways involving AMPK [9].

### Kinase assays

AMPK kinase assays were performed using full-length recombinant proteins in 50 mM HEPES (pH 7.4), 1 mM AMP, 1 mM (γ-^32^P) ATP, 5 mM MgCl_2_, 1 pM AMPK (Sigma Aldrich), and the putative target recombinant protein (1 nM) in a 50 μl reaction volume at 37°C for 1 h. Proteins were then resolved by using SDS-PAGE, stained with Coomassie blue, and subjected to autoradiographic analysis. Peptide assays were performed under the same conditions but with 3 nM peptide, (γ-^32^P) ATP 1 mM, and 1 pM AMPK. SAMS peptide (3 nM) was used as positive control. Peptide reactions were terminated by blotting samples onto Whatman grade P81 ion-exchange chromatography paper, rinsed in 1% phosphoric acid, washed in acetone, and allowed to air dry prior to submersion in 1 ml scintillation fluid. The radioactivity was measured with a Beckman LS 6500 scintillation counter.

### Cell lines

MEF cells were isolated from mice according to standard protocols [58]. HUVEC and C2C12 cells were purchased from American Type Culture Collection (ATCC) Cat# CRL-1730 and CRL-1772 respectively.

### siRNA knockdown of gene expression

siRNA knockdown was conducted according to standard protocols. The following human siRNA sequences were used from Qiagen: Scrambled, AMPK, AKT2, and ATF2, Cat# 1022076, SI00086359, SI00305872, SI00305872 respectively. The following mouse siRNA sequences were used from Qiagen: AMPK, FOXO3a, and NADSYN1, cat# SI01388219, SI01005207, and GS78914 respectively.

### Chromatin immunoprecipitation (ChIP) assay

Cells were incubated in 0.75% paraformaldehyde and harvested in FA lysis buffer (50 mM HEPES-KOH, pH 7.5, 140 mM NaCl, 1 mM EDTA, 1% Triton X-100, 0.1% sodium deoxycholate, 0.1% SDS and Halt Protease inhibitors) prior to sonication with a Bioruptor 200. Protein was immunoprecipitated overnight at 4°C, washed with wash buffer (0.1% SDS, 1% Triton X-100, 2 mM EDTA, pH 8.0, 150 mM NaCl, 20 mM Tris–HCl (pH 8.0) and eluted with 100 μl elution buffer (1% SDS, 100 mM NaHCO_3_). DNA was purified using Qiagen PCR purification kit prior to qPCR analysis. The following primers were used for qPCR analysis of immunoprecipitated DNA: NADSYN1 promoter forward, ATTCCTTGGCTTCCTACTGC; NADSYN1 promoter reverse, GTGTCTTGATAGATGGGCTACAG; MMP-2 promoter forward, GCAGAAGGAAAGAGGTAAGGAAG; MMP-2 promoter reverse, GAAGGAATGGTCAGAAACAGATG.

### Quantitative real-time PCR (qRT-PCR)

RNA was purified using TRIzol reagent from Life Technologies. Two ng of RNA was converted to cDNA using Promega reverse transcriptase according to the manufacturer’s instructions. cDNA was then quantified via qPCR using SYBER green qPCR master mix from Bio-Rad. Results were calculated using the delta-delta ct method. The following primers were used for qRT-PCR analysis: NADSYN1 forward, GAGAGCCTTTGTCCAGTTTTG; NADSYN1 reverse, GTTGTCATCTTGTGCCTGTTC. MMP-2 forward, TTGAGAAGGATGGCAAGTACG; MMP-2 reverse, TGGTGTAGGTGTAAATGGGTG.

### Immunoblotting

Cells were lysed in 10 mM Tris (pH 7.4), 0.1 M NaCl, 1 mM EDTA, 1 mM EGTA, 1 mM NaF, 20 mM Na_4_P_2_O_7_, 2 mM sodium orthovanadate (Na_3_VO_4_), 0.1% SDS, 0.5% Sodium Deoxycholate, 1% Triton X-100, 10% Glycerol, 1 mM PMSF and protease inhibitors. The obtained cell lysates were then resolved by 10% SDS-PAGE, blocked with 5% milk, rinsed with TBST, probed with NADSYN, FOXO3a, β-actin, AMPK, MMP2, ATF2 primary antibodies from ABcam cat# ab139561, ab12162, ab8227, ab110036, ab86607, and ab47476 respectively, P-FOXO3a and P-ATF2 primary antibodies from Cell Signaling (Cat # 8174 and 9221), and secondary antibodies (Cat# 7074S and 7076S). The blotted proteins bands were made detectable by Emerson chemiluminescence substrate (ECL) and image captured on HyBlot CL autoradiography film.

### MMP-2 assay

MMP-2 activity was determined using Amersham Matrix Metalloproteinase-2 (MMP-2) Biotrak Activity Assay System RPN2631 protocol.

### NADSYN1 assay

The cellular concentration of NAD^+^ was measured in C2C12 cell lysates using EnzyChrom^TM^ NAD^+^/NADH Assay Kit (E2ND-100) and protocol from BioAssay Systems.

### Statistical analysis

Data are expressed as means ± SEM of at least three independent experiments. Comparisons of mean values between two groups were evaluated using a two-tailed Student’s t-test, Wilcoxon signed-rank test or Mann–Whitney U test. Unless otherwise indicated, *p < 0.05 was considered statistically significant.

## Additional Files

Additional file 1: Table S1. Putative AMPK substrates. Additional file 1: Table S1 contains a representative list of putative AMPK substrates along with their official symbols, official names, cellular locations, and functions. Additional file 2: Figure S1. Predicted modules of AMPK phosphorylated substrates and the related functions. **(A)** AMPK phosphorylation of ARRB1 inhibits GPCR signaling and increases histone acetylation activity at c-Fos and p27 promoters. **(B)** AMPK phosphorylation of DDX56 TOP gene translation and subsequent 60S ribosomal biogenesis. **(C)** AMPK phosphorylation of Calnexin acts a chaperone to positively regulate ER Ca^2+^ efflux through SERCA2b. **(D)** AMPK phosphorylation of HMGA1 increases INSR and Glut3 transcription through the Cav-1/AMPK pathway. **(E)** AMPK phosphorylation of KLF2 increases LP4R transcription and SMC recruitment. **(F)** AMPK phosphorylation of Treacle associates with UBF to increase AMPK-related gene transcription. **(G)** AMPK phosphorylation of CHMP1b facilitates lysosomal recycling of proteins and increases S1 cell cycle arrest. **(H)** AMPK phosphorylation of CPSF2 increases translation of AMPK related genes. **(I)** AMPK phosphorylation of NUCKS transcriptionally regulates the circadian rhythm. **(J)** AMPK phosphorylation of ZRF1 inhibits ATPase activity but increases transcription of AMPK-related genes.
