# Supplementary material for: Identification of AMP-activated protein kinase targets by a consensus sequence search of the proteome
Source: BMC Syst Biol. 2015 Mar 11;9:13. doi: 10.1186/s12918-015-0156-0 (PMC4357066; doi:10.1186/s12918-015-0156-0)
Supplement: Additional file 1: Table S1. — Putative AMPK substrates. Additional file 1: Table S1 contains a representative list of putative AMPK substrates along with their official symbols, official names, cellular locations, and functions. [file 12918_2015_156_MOESM1_ESM.pdf]

Additional Table 1: Representative putative AMPK substrates

Data specifics obtained from http://www.ncbi.nlm.nih.gov/gene

Official Symbol	Official Full Name	Location	Function
A2M	alpha-2-macroglobulin	extracellular space	response to nutrient; response to glucocorticoid stimulus; response to carbon dioxide; negative regulation of complement activation, lectin pathway
ABCA1	ATP-binding cassette, sub-family A (ABC1), member 1	cell/plasma membrane; Golgi apparatus; Golgi apparatus	lysosome organization; negative regulation of macrophage derived foam cell differentiation; phagocytosis, engulfment; positive regulation of cAMP
ABCB1	ATP-binding cassette, sub-family B (MDR/TAP), member 1	cell membrane	ion transmembrane transport
ACACA	acetyl-CoA carboxylase alpha	cytoplasm; nucleus	lipid metabolism; fatty acid syntheis; acetyl-CoA metabolic process
ACADS	acyl-CoA dehydrogenase, C-2 to C-3 short chain	mitochondrion	response to starvation; oxidation reduction; response to glucocorticoid stimulus; lipid metabolism; fatty acid metabolic process; fatty acid beta oxidation
ACIN1	apoptotic chromatin condensation inducer 1	cytoplasm; nucleus	apoptosis; positive regulation of ; monocyte differentiation; apoptotic chromosome condensation; erythrocyte differentiation
ACOT7	acyl-CoA thioesterase 7	cytoplasm; mitochondrion	fatty acid catabolic process; lipid metabolic process
ACOT8	acyl-CoA thioesterase 8	mitochondrion; peroxisome	fatty acid beta-oxidation using acyl-CoA oxidase; lipid metabolic process; peroxisome organization
ACSL3	acyl-CoA synthetase long-chain family member 3	endoplasmic reticulum; membrane; microsome; mitochondrial membrane; nucleus; peroxisome	fatty acid biosynthetic process
ACTA2	actin, alpha 2, smooth muscle, aorta	cytoplasm; cytoskeleton	virus integration/response to virus; lipid metabolic process
ACTC1	actin, alpha, cardiac muscle 1	actin; cytoplasm; cytoskeleton	actomyosin structure organization; response to ethanol; apoptosis
ACTL6A	actin-like 6a	nucleus; plasma membrane	chromatin remodeling; transcription; Histone H2A and H4 acetylation; regulation of growth; signal transduction
ACTN1	actinin, alpha 1	cytoskeleton; cytoplasm; myofibril; nucleus; sarcomere; Z-disk	regulation of apoptosis; focal adhesion assembly; negative regulation of cellular component movement
ACTN4	actinin, alpha 4	actin cytoskeleton; cytoplasm; nucelus	actin filament bundle assembly; positive regulation of pinocytosis; positive regulation of sodium:hydrogen antiporter activity; protein transport; regulation of apoptosis; response to hypoxia
ADD1	adducin 1 (alpha)	cytosol; membrane; nucleus	actin cytoskeleton organization; actin filament bundle assembly; barbed-end actin filament capping
ADNP	activity-dependent neuroprotector homeobox	axon; cytoplasm; dendrite; extracellular space; neuronal cell body; nucleus	negative regulation of gene expression; negative regulation of neuron apoptosis; negative regulation of synaptic transmission

AHNAK	AHNAK nucleoprotein	actin cytoskeleton; cell-cell contact zone; costamer; cytoplasm; lysosomal memebrane; nucleus; sarcolemma; vesicle	S100 protein binding; poly (A) RNA binding; structural molecular activity conferring elasticity
AIFM1	apoptosis-inducing factor, mitochondrion-associated, 1	cytoplasm; microsome; mitochondrion; nucleus	DNA damage response, signal transduction resulting in induction of apoptosis/DNA fragmentation involved in apoptotic nuclear change/neuron apoptosis
AIMP1	aminoacyl tRNA synthetase complex-interacting multifunctional protein 1	cytoplasm;extracellular space; endoplasmic reticulum; gogli apparatus; nucleus	angiogenesis; apoptosis; carbohydrate metabolic process; cell adhesion; cell - cell signaling; glucose metabolic process; inflammatory response
AIP	aryl hydrocarbon receptor interacting protein	cytoplasm	xenobiotic metabolic process; protein maturation by protein folding
AKR1B10	aldo-keto reductase family 1, member B10 (aldose reductase)	cytoplasm	oxidation reduction; cellular aldehyde metabolic process; steroid metabolic process
AKT2	v-akt murine thymoma viral oncogene homolog 2	mitochondrion; nucleus; plasma membrane	regulation of JNK cascade; positive regulation of gene expression; glucose metabolic process; positive regulation of positive chemotaxis
ALDH18A1	aldehyde dehydrogenase 18 family, member A1	cytoplasm; mtochondrion inner membrane	oxidation reduction; proline biosynthetic process
ALDH7A1	aldehyde dehydrogenase 7 family, member A1	cytoplasm; mitochondria; nucleus	cellular aldehyde metabolic process; oxidation reduction; sensory perception of sound
AMPD1	adenosine monophosphate deaminase 1	undefiend	nucleotide metabolic process; purine base metabolic process/purine ribonucleoside monophosphate biosynthetic process; extracellular matrix organization
ANAPC1	anaphase promoting complex subunit 1	cytoplasm; nucleus; plasma membrane	cellular component movement; anaphase-promoting complex-dependent proteasomal ubiquitin-dependent protein catabolic process; cell cycle
APBB2	amyloid beta (A4) precursor protein-binding, family B, member 2	nucelus	cell cycle arrest; regulation of transcription; negative regulation of cell growth; negative regulation of S phase of mitotic cell cycle
APBB3	amyloid beta (A4) precursor protein-binding, family B, member 3	actin cytoskeleton; cytoplasm	beta-amyloid binding; protien binding; trasncription factor binding
APEX1	APEX nuclease (multifunctional DNA repair enzyme) 1	cytoplasm; endoplasmic reticulum; nucleus	cell redox homeostasis; transcription from RNA polymerase II promoter; base-excision repair
APOA1BP	apolipoprotein A-I binding protein	extracellular space; mitochondrion	NADHX epimerase activity; metal ion binding; nucleotide binding; protein binding; protein homodimeraization activity
APRT	adenine phosphoribosyltransferase	cytoplasm	purine ribonucleoside salvage; adenine metabolic process; adenine salvage; nucleoside metabolic process
ARHGAP5	rho GTPase activating protein 5	cytoplasm; membrane	signal transduction; cell adhesion; small GTPase mediated signal transduction; Rho protein signal transduction; positive regulation of cell migration; positive regulation of mesenchymal cell proliferation; regulation of cell size
ARHGEF1	rho guanine nucleotide exchange factor (GEF) 1	cytoplasm; plasma membrane	Rho protein signal transduction; Cell proliferation; negative regulation of axonogenesis; positive regulation of axonogenesis

ARL2	ADP-ribosylation factor-like 2	cytoplasm; goli apparatus	cell cycle; small GTPase mediated signal transduction; tubulin complex assembly; acetylcholine transport
ARRB1	arrestin, beta 1	cytoplasm; chromatin; cytoplasmic membrane -bounded vesicle; nucleus	G-protein coupled receptor internalization; cellular membrane organization; histone H4 acetylation
ASCC3	activating signal cointegrator 1 complex subunit 3	cytoplasm	transcription
ATG5	ATG5 autophagy related 5 homolog (S. cerevisiae)	cytoplasm	negative regulation of apoptosis; negative regulation of protein ubiquitination; autophagic vacuole assembly; autophagy
ATP1B1	ATPase, Na+/K+ transporting, beta 1 polypeptide	plasma membrane	potassium ion transport; response to hypoxia; sodium ion transport; ATP biosynthetic process
ATP2A2	ATPase, Ca++ transporting, cardiac muscle, slow twitch 2	endoplasmic reticulum; microsome; nucleus; sarcoplasmic reticulum membrane	ATP biosynthetic process; cation transport; calcium ion homeostasis; cell adhesion; ER-nucleus signaling pathway
ATP2A3	ATPase, Ca++ transporting, ubiquitous	endoplasmic reticulum; nucleus; sarcoplasmic reticulum membrane	calcium ion transport; cation transport; ATP biosynthetic process
ATRX	alpha thalassemia/mental retardation syndrome X-linked	chromatin; nucleus	DNA methylation; DNA recombination; transcription
ATXN2	ataxin-2	cytoplasm; golgi apparatus	RNA transport; cell death; regulation of translation; cytoplasmic mRNA processing body assembly
AVIL	advillin	cytoplasm; cytoskeleton	response to stress ; cytoskeleton organization
BAG1	BCL2-associated athanogene	cytoplasm; nucleus	apoptosis; cell surface receptor linked signaling pathway; chaperone cofactor-dependent protein refolding; anti-apoptosis
BAI1	brain-specific angiogenesis inhibitor 1	cell plasma membrane	axonogenesis; cell adhesion; negative regulation of cell proliferation; signal transduction; G-protein coupled receptor protein signaling pathway
BAK1	BCL2-antagonist/killer 1	cytoplasm; mitochondrion outer membranee	regulation of apoptosis; establishment or maintenance of transmembrane electrochemical gradient
BANF1	barrier to autointegration factor 1	cytoplasm; nucleus	virus integration/response to virus
BAT2	HLA-B associated transcript 2	cytoplasm; nucleus	inflammation
BAZ1B	bromodomain adjacent to zinc finger domain protein 1B	nucleus	DNA ligation or repair or damage response; Histone modification/phosphorylation; nucleosome assembly (on/off); transcription; regulation of gene expression/epigenetics
BBS2	Bardet-Biedl syndrome 2	cell projection; cytoplasm; plasma membrane	fat cell differentiation; photoreceptor cell maintenance; protein localization; regulation of cilium beat frequency involved in ciliary motility; sperm axoneme assembly

BCL11A	B-cell CLL/lymphoma 11A (zinc finger protein)	cytoplasm; nucleus	hemopoiesis; protein sumoylation; regulation of transcription
BCL9	B-cell CLL/lymphoma 9	nucleolus; nucleus	regulation of canonical Wnt receptor signaling pathway
BCLAF1	BCL2-associated transcription factor 1	nucleus; cytoplasm	induction of apoptosis; negative regulation of transcription; positive regulation of apoptosis
BGN	biglycan	extracellular space and matrix	blood vessel remodeling; peptide cross-linking via chondroitin 4-sulfate glycosaminoglycan
BMS1B	BMS1 ribosome biogenesis factor	nucleolus; nucleus	ATP binding; poly (A) RNA binding
BNIP2	BCL2/adenovirus E1B 19kDa interacting protein 2	cytoplasm;nuclear membrane/envelope; cell/plasma membrane	anti-apoptosis
BRAF	v-raf murine sarcoma viral oncogene homolog B1	cytoplasm; synaptosome; cell/plasma membrane; nucleus	MAPKKK cascade; negative regulation of neuron apoptosis; positive regulation of ERK1 and ERK2 cascade; protein amino acid phosphorylation
BRD3	bromodomain containing 3	nucelus	chromatin binding; lysine-acetylated histone binding; protein binding
BRD4	bromodomain containing 4	condensed nuclear chromsome; cytoplasm; nucleoplasm; nucleus; positive transcription factor elogantion factor complex b	DNA binding; chromatin binding; lysine-acetylated histone binding; p53 binding; protein binding
BSG	basigin	melanosome; embrane	cell surface receptor linked signaling pathway; decidualization; odontogenesis of dentine-containing tooth; response to cAMP; response to mercury ion; response to peptide hormone stimulus
BTF3	basic transcription factor 3	nucleus	transcription; transcription from RNA polymerase II promoter
C3	complement component 3	extracellular space	inflammatory response; positive regulation of phagocytosis; signal transduction; positive regulation of angiogenesis; immune response
CALR	calreticulin	cytoplasm; endoplasmic reticulum; extracellular space; microsome; nucleus; plasma membrane	cell cycle arrest; cellular calcium ion homeostasis; cortical actin cytoskeleton organization; glucocorticoid receptor signaling pathway
CAMK2G	calcium/calmodulin-dependent protein kinase II gamma	cytosol; endocytic vescicle membrane; membrane; nucleoplasm; sarcoplasmic reticulum membrane	ATP binding; calcium-dependent protein serine/threonine phosphatase activity; calmodulin binding
CANX	calnexin	axon; dendrite cytoplasm and spine; endoplasmic reticulum; melanosome; melanosome; neuronal cell body; ribosome	apoliprotein binding; calcium ion binding; carbohydrate binding; glycoprotein binding; inotropic glutamate receptor binding; poly (A) RNA binding; unfolded protein binding
CAPN2	calpain 2, (m/II) large subunit	cytoplasm; cell membrane; plasma membrane	proteolysis; response to hypoxia; myoblast fusion
CARS	cysteinyl-tRNA synthetase	cytoplasm	cysteinyl-tRNA aminoacylation; translation

CASP8	caspase 8, apoptosis-related cysteine peptidase	cytoplasm;mitochondrion; nucleus	angiogenesis; apoptosis; macrophage differentiation; positive regulation of I-kappaB kinase/NF-kappaB cascade; protein heterooligomerization
CAST	calpastatin	undefined	blocks TNF alpha induced NfKappa beta; proteolysis
CAV1	caveolin 1, caveolae protien	caveola; cytoplasm; endoplasmic reticulum; golgi apparatus; plasma membrane	calcium homeostasis; caveolae formation and stabilization; cholesterol homeostasis; cellular response to starvation
CBX5	chromobox protein homolog 5	centrosome; chromatin; cytoplasm; nucleus	chromatin remodeling; Negative regulation of transcription
CCNB1	cyclin B1	centrosome; cytoplasm; nucleus	cell division; cellular response to hypoxia; mitotic spindle stabilization; negative regulation of gene expression
CCT4	chaperonin containing TCP1, subunit 4 (delta)	cytoplasm; melasome	protein folding
CCT7	chaperonin containing TCP1, subunit 7 (eta)	cytoplasm; mitochondrion	Protein folding
CD2BP2	CD2 (cytoplasmic tail) binding protein 2	cytoplasm; nucleus; nucleoplasm	RNA splicing
CDAN1	congenital dyserythropoietic anemia, type I	cytoplams; endomembrane system; integral component of membrane; nucleus; plasma membrane	protien binding
CDC37	cell division cycle 37 homolog	cytoplasm	regulation of cyclin-dependent protein kinase activity; regulation of interferon-gamma-mediated signaling pathway/ type I interferon-mediated signaling pathway
CDC42BPB	Cdc42 binding protein kinase beta	cytoplasm; cell/plasma membrane	signal transduction; phosphorylation
CDK111	cyclin-dependent kinase 11A	cytoplasm; nucleus	apoptosis; regulation of cell growth; regulation of transcription, DNA-dependent; regulation of mRNA processing; mitosis
CDK2	cyclin-dependent kinase 2	cytoplasm; nucleus	DNA replication; G2/M transition of mitotic cell cycle; Ras protein signal transduction; cell division; Mitosis; positive regulation of cell proliferation
CFDP1	craniofacial development protein 1	cytoplasm; nucleus	apoptosis(anti); cell adhesion
CHD4	chromodomain helicase DNA binding protein 4	chromatin; nucleus	transcription
CHD5	chromodomain helicase DNA binding protein 5	chromatin; nucleus	transcription
CHERP	calcium homeostasis endoplasmic reticulum protein	cytoplasm; cytoskeleton; endoplasmic reticulum	mRNA processing

CHMP1B	charged multivesicular body protein 1B	cytosol; extracellular vesicular exosome; late endosome membrane	protein binding; protein domain specific binding
CIT	citron (rho-interacting, serine/threonine kinase 21)	cytoplasm	cell cycle; cell differentiation; cell division; mitosis; protein amino acid phosphorylation
CKMT1A	creatine kinase, mitochondrial 1A	membrane; mitochondria	creatine metabolic process
CLASP1	cytoplasmic linker associated protein 1	centrosome; chromatin; cytoplasm; cytoskeleton; golgi apparatus; kinetochore	cell cycle; cell division; establishment or maintenance of cell polarity; exit from mitosis; microtubule bundle formation
CNN2	calponin 2	cell to cell junction; cytoskeleton	cytoskeleton organization; regulation of actin filament-based process; actomyosin structure organization
COPB1	coatomer protein complex, subunit beta 1	cytoplasm; golgi apparatus membrane; peripheral membrane protein	intracellular protein transport; vesicle-mediated transport; COPI coating of Golgi vesicle; cellular membrane organization
COPS2	COP9 constitutive photomorphogenic homolog subunit 2	cytoplasm; nucleus	signal transduction; transcription from RNA polymerase II promoter
CORO1B	coronin, actin binding protein, 1B	actin cytoskeleton; actin filament; cell leading edge; cytoplams; lamellipodium; plasnma membrane; stress fiber	Arp2/3 complex binding; actin filament binding
CORO1C	coronin, actin binding protein, 1C	actin cytoskeleton	phagocytosis; signal transduction
COX	cytochrome c oxidase	mitochondrion inner membrane	cytochrome-c oxidase activity; elctron carrier activity; metal ion binding
CPD	carboxypeptidase D	microsome; nucelus	proteolysis
CPNE2	copine II	membrane	calcium-dependent binding
CPOX	coproporphyrinogen oxidase	cytoplasm; mitochondrion intermembrane space	oxidation reduction; response to mercury ion; response to arsenic; heme biosynthetic process
CPSF2	cleavage and polyadenylation specific factor 2, 100kDa	nucleus	RNA splicing; histone mRNA 3'-end processing
CRISP1	epididymal secretory protein E1	extracellular space	fusion of sperm to egg plasma membrane
CROT	carnitine O-octanoyltransferase	perioxosome	fatty acid metabolic process; lipid metabolic process; transport
CRYZL1	crystallin, zeta (quinone reductase)-like 1	cytoplasm	oxidation reduction; quinone cofactor metabolic process

CTNNBL1	catenin, beta like 1	nucleus	positive regulation of apoptosis; somatic diversification of immunoglobulins
CTNND1	catenin (cadherin-associated protein), delta 1	cytoplasm; nucleus; plasma membrane	Wnt receptor signaling pathway; cell adhesion; epithelial cell differentiation involved in salivary gland development; regulation of transcription
CTTN	cortactin	cytoplasm; cytoskeleton; lamellipodium	actin assembly; regulation of endocytosis
CUL3	cullin 3	nucelus; Golgi apparatus	G1/S transition of mitotic cell cycle; cell cycle arrest; cell migration; cyclin catabolic process; protein ubiquitination
CUL4B	cullin 4B	nucleus	DNA repair; cell cycle; ubiquitin-dependent protein catabolic process
CUL5	cullin 5	cytoplasm; membrane; nucleus	G1/S transition of mitotic cell cycle; cell cycle arrest; cytosolic calcium ion homeostasis; induction of apoptosis by intracellular signals
CUL7	cullin 7	cytolplasm	proteolysis; regulation of mitotic metaphase/anaphase transition; ubiquitin-dependent protein catabolic process; vasculogenesis
CYSLTR2	cysteinyl leukotriene receptor 2	cell membrane	immune response
DAB2	disabled homolog 2, mitogen-responsive phosphoprotein	cytoplasmic vesicle; membrane; nucleus	cell morphogenesis involved in differentiation; pinocytosis; cell proliferation; negative regulation of cell growth
DARS	aspartyl-tRNA synthetase	cytoplasm	aspartyl-tRNA aminoacylation; translation
DBN1	drebrin 1	actin cytoskeleton; cytoplasm	actin filament organization; cell differentiation; regulation of neuronal synaptic plasticity
DDIT3	growth arrest and DNA-damage-inducible protein GADD153	cytoplasm; nucleus	cell redox homeostasis; cell cycle; positive regulation of transcription; positive regulation of apoptosis; response to oxidative stress
DDOST	dolichyl-diphosphooligosaccharide--protein glycosyltransferase	endoplasmic reticulum membrane	T cell activation; response to cytokine stimulus; protein amino acid terminal N-glycosylation
DDX56	DEAD (Asp-Glu-Ala-Asp) box polypeptide 56	nucleolus; membrane	ATP binding; Atp-dependenthelicase activity; ATPase activity; poly (A) RNA binding; protein binding
DGCR8	DiGeorge syndrome critical region gene 8	cytoplasm; nucleus	primary microRNA processing
DGKA	diacylglycerol kinase, alpha 80kDa	cytoplasm; plasma membrane	activation of protein kinase C activity by G-protein coupled receptor protein signaling pathway; signal transduction
DHRS4	dehydrogenase/reductase (SDR family) member 4	mitochondrion; peroxisome	oxidation reduction

DHX9	DEAH (Asp-Glu-Ala-His) box polypeptide 9	cytoplasm; nucleus	RNA splicing; cellular response to heat; CRD-mediated mRNA stabilization
DIP2B	DIP2 disco-interacting protein 2 homolog B (Drosophila)	cytoplasm; extracellular vesciular exosome; membrane; nucleus	catalytic activity; molecular function; transcription factor binding
DIS3	DIS3 mitotic control homolog	cytoplasm; nucleus	rRNA processing/transcription
DKC1	dyskeratosis congenita 1, dyskerin	nucleolus; nucleus	rRNA processing/transcription; cell proliferation; pseudouridine synthesis; pseudouridine synthesis; telomere maintenance via telomerase
DLGAP4	discs, large (Drosophila) homolog-associated protein 4	golgi apparatus; cell/plasma membrane; synapse	cell-cell signaling
DNAJC8	DnaJ (Hsp40) homolog, subfamily C, member 8	not well defined	RNA splicing
DNASE2	deoxyribonuclease II, lysosomal	lysosome	apoptosis; erythrocyte differentiation
DNM1	dynamin 1	cytoplasm; cytoskeleton; microtubule	endocytosis
DNMT1	DNA (cytosine-5-)-methyltransferase 1	cytoplasm; nucleus	DNA methylation; chromatin modification; negative regulation of histone H3-K9 methylation; positive regulation of gene expression
DNPEP	aspartyl aminopeptidase	cytoplasm	peptidase; Proteolysis
DOCK7	dedicator of cytokinesis 7	cell projection	axonogenesis; cell differentiation; microtubule cytoskeleton organization; positive regulation of peptidyl-serine phosphorylation; activation of Rac GTPase activity; establishment of neuroblast polarity
DPF3	D4, zinc and double PHD fingers, family 3	nucleus	chromatin modification; regulation of transcription
DPYSL2	dihydropyrimidinase-like 2	cytoplasm; mitochondrion	cell differentiation; nucleobase, nucleoside, nucleotide and nucleic acid metabolic process; positive regulation of glutamate secretion
DRAP1	DR1-associated protein 1 (negative cofactor 2 alpha)	nucleus	regulation of transcription
DRG1	developmentally regulated GTP binding protein 1	cytoplasm; nucleus	transcription
DRG2	developmentally regulated GTP binding protein 2	cytoplasm; mitochondrion	signal transduction
DVL1	dishevelled, dsh homolog 1	cytoplasm	Wnt receptor signaling pathway; neurotransmitter secretion; signal transduction; Transcription; synapse organization; protein localization in nucleus

DYNLRB1	dynein, light chain, roadblock-type 1	cytoplasm	microtubule-based movement; visual behavior
EEA1	early endosome antigen 1	cytoplasm; early endosome; plasma membrane	synaptic vesicle to endosome fusion; early endosome to late endosome transport
EEF1B2	eukaryotic translation elongation factor 1 beta 2	cytoplasm	translation
EEF1G	eukaryotic translation elongation factor 1 gamma	cytosol	response to virus; translational elongation
EEF2K	eukaryotic elongation factor-2 kinase	nucleus; cytoplasm	protein amino acid phosphorylation; translational elongation
EEFSEC	eukaryotic elongation factor, selenocysteine-tRNA-specific	cytoplasm; nucleus; mitochondrion	translational elongation; selenocysteine incorporation
EGFR	epidermal growth factor receptor	cytoplasm; endosome; extracellular space; nucleus; plasma membrane	activation of MAPKK activity; cell-cell adhesion; ossification; epidermal growth factor receptor signaling pathway; cerebral cortex cell migration
EHMT2	euchromatic histone-lysine N-methyltransferase 2	nucleus	chromatin modification
EIF1AX	eukaryotic translation initiation factor 1A, X-linked	cytoplasm	translational initiation
EIF1B	eukaryotic translation initiation factor 1B	cytoplasm	poly (A) RNA binding; translation intiation factor activiy
EIF2AK2	eukaryotic translation initiation factor 2-alpha kinase 2	not well defined	endoplasmic reticulum unfolded protein response; negative regulation of cell proliferation
EIF2B2	eukaryotic translation initiation factor 2B, subunit 2 beta, 39kDa	cytoplasm	response to heat; translational initiation; response to glucose stimulus; myelination; response to peptide hormone stimulus
EIF2B5	eukaryotic translation initiation factor 2B, subunit 5 epsilon, 82kDa	cytoplasm; nucleus	astrocyte differentiation; response to heat; response to glucose stimulus; response to peptide hormone stimulus; regulation of translation; response to stress; myelination
EIF2C1	eukaryotic translation initiation factor 2C, 1	cytoplasm; nucleus	gene silencing by RNA; negative regulation of translation involved in gene silencing by miRNA; nuclear-transcribed mRNA catabolic process; regulation of transcription
EIF2C3	eukaryotic translation initiation factor 2C, 3	cytoplasm; P-body	gene silencing by RNA; mRNA catabolic process; negative regulation of translation involved in gene silencing by miRNA
EIF3	ıkaryotic translation initiation factor 4/	cytosol; eukaryotic translation initaiton factor 4F complex; perinuclear region of cytoplasm	ATP binding; Atp-dependenthelicase activity; ATPase activity; poly (A) RNA binding; protein binding; translation intitiaton factor activity
EIF4A2	eukaryotic translation initiation factor 4A2	cytosol; eukaryotic translation initaiton factor 4F complex; perinuclear region of cytoplasm	ATP binding; Atp-dependenthelicase activity; ATPase activity; poly (A) RNA binding; protein binding; translation intitiaton factor activity

EIF5A	eukaryotic translation initiation factor 5A	cytoplasm; endoplasmic reticulum; nucleus	apoptosis; translation; protein transport; positive regulation of cell proliferation; mRNA export from nucleus
EIF5B	eukaryotic translation initiation factor 5B	cytosol	GTP binding; GTPase activit; poly (A) RNA binding; protein binding; translation intitiaton factor activity
ELMO1	engulfment and cell motility 1	cytoplasm; nucleus; plasma membrane	apoptosis; Rac protein signal transduction; phagocytosis, engulfment; actin cytoskeleton organization
ENO1	enolase 1, (alpha)	cytoplasm; nucleus; plasma membrane	glycolysis; negative regulation of cell growth; negative regulation of transcription from RNA polymerase II promoter; negative regulation of transcription, DNA-dependent; response to virus
ENO3	enolase 3 (beta, muscle)	cytoplasm	glycolysis
ENSA	endosulfine alpha	cytolplasm	response to nutrient
EPB41	erythrocyte membrane protein band 4.1 (elliptocytosis 1, RH-linked)	cytoplasm; nucleus; plasma membrane	actin cytoskeleton organization
EPPK1	epiplakin 1	cytoplasm; cytoskeleton	poly (A) RNA binding
EPS8	epidermal growth factor receptor pathway substrate 8	synaptosome	actin cytoskeleton reorganization; cell proliferation; response to ethanol; epidermal growth factor receptor signaling pathway; signal transduction
ERCC8	excision repair cross-complementing rodent repair deficiency, complementation group 8	nuclear membrane; nucleus	nucleotide-excision repair/transcription coupled; response to oxidative stress; positive regulation of DNA repair
ERO1L	ERO1-like protein alpha	endoplasmic reticulum membrane; peripheral membrane protein	brown fat cell differentiation; electron transport chain; endoplasmic reticulum unfolded protein response; protein thiol-disulfide exchange
ESF1	nucleolar pre-rRNA processing protein	nucleolus; nucleus	poly (A) RNA binding
ETFB	electron-transfer-flavoprotein, beta polypeptide	mitochondrial matrix	electron transport chain
ETNK2	ethanolamine kinase 2	undefined	phosphatidylethanolamine biosynthetic process; phospholipid biosynthetic process
EXOSC3	exosome component 3	cytoplasm; nucleus	rRNA processing
FAF1	Fas (TNFRSF6) associated factor 1	cytoplasm; nucleus	cell death; positive regulation of apoptosis; regulation of cell adhesion; proteasomal ubiquitin-dependent protein catabolic process; cytoplasmic sequestering of NF-kappaB; regulation of protein catabolic process
FAM129B	family with sequence similarity 129, member B	actin cytoskeleton; adherens junction; cytoplasm; cytosol; ectracellular vascular exosome; nucleolus; nucleoplasm; nucleus; plasma membrane	negative regulation of apoptosic process

FANCI	fanconi anemia, complementation group I	cytoplasm; nucleus	DNA repair; Cell cycle
FARP2	FERM, RhoGEF and pleckstrin domain protein 2	cytoplasm; cell/plasma membrane	Rac protein signal transduction; neuron remodeling; regulation of Rho protein signal transduction
FASN	fatty acid synthase	cytoplasm; golgi apparatus; melasome; mitiochondrion	fatty acid biosynthetic process; fatty acid metabolic process; oxidation reduction
FBXL15	F-box and leucine-rich repeat protein 15	SCF ubiquitin complex; cytoplasm	protein binding; contributes to ubiquiti-protein transferase activity
FBXO38	F-box protein 38	cytoplasm; nucleus	undefined
FBXO8	F-box protein 8	cell junction; nucleus; not trans-Golgi network; ubiquitin ligase complex	regulation of ARF protein signal transduction; ubiquitin-dependent protein catabolic process
FKBP4	FK506 binding protein 4, 59kDa	cytoplasm	androgen receptor signaling pathway; copper ion transport; negative regulation of microtubule polymerization or depolymerization
FLNA	filamin A, Alpha-filamin	actin; cytoplasm; cytoskeleton; extracellular space; myofibril;nucleus; plasma membrane; trans-golgi network	actin cytoskeleton reorganization; early endosome to late endosome transport; establishment of protein localization
FLNB	filamin B, beta	actin; cytoplasm; cytoskeleton; myofibril; plasma membrane	actin cytoskeleton organization; cell differentiation; signal transduction
FMR1	fragile X mental retardation 1	cytoplasm	mRNA transport; negative regulation of translational initiation
FNBP4	formin binding protein 4	nucleoplasm	protein binding
FTH1-A	ferritin, heavy polypeptide 1	cytoplasm; extracellular vescicular exosome; intracellular ferritin complex; mitochondrion; nucleus	ferric ion binding; ferroxidase activity; iron ion binding; protein binding
FTSJD2	FtsJ methyltransferase domain containing 2	nucleus	methyltransferase
FUBP1	far upstream element (FUSE) binding protein 1	nucleus	regulation of transcription
Fus	fus fusion, derived from t(12;16) malignant liposarcoma	cytoplasm; nucleus	angiogenesis
FXR1	fragile X mental retardation, autosomal homolog 1	cytoplasm; nucleolus; nucleus	apoptosis; negative regulation of translation; cell differentiation
GAA	glucosidase, alpha; acid	lysosome	carbohydrate metabolic process

GABARAP	gamma-aminobutyric acid receptor associated protein	cytoplasm; cytoskeleton; endoplasmic reticulum; golgi apparatus membrane	microtubule cytoskeleton organization; protein transport; synaptic transmission; protein targeting
GABRB1	gamma-aminobutyric acid (GABA) A receptor, subunit beta 1	synapse; cell/plasma membrane	ion transport; signal transduction
GANAB	glucosidase, alpha; neutral AB	endoplasmic reticulum; golgi apparatus; melanosome	carbohydrate metabolic process
GAPDH	glyceraldehyde-3-phosphate dehydrogenase	cytoplasm; cell/plasma membrane	glucose metabolism/glycolysis; oxidation reduction
GARS	glycyl-tRNA synthetase	cytoplasm; mitochondrion	cell death; diadenosine tetraphosphate biosynthetic process; glycyl-tRNA aminoacylation
GART	phosphoribosylglycinamide formyltransferase	cytoplasm	biosynthetic process
GCC2	GRIP and coiled-coil domain-containing protein 2	cytoplasm; golgi apparatus membrane; peripheral membrane protein	late endosome to Golgi transport/protein localization of golgi and lysosome; retrograde transport, endosome to Golgi
GDAP1	ganglioside-induced differentiation associated protein	cytoplasm	response to retinoic acid
GFM1	G elongation factor, mitochondrial 1	mitochonrian	mitochondrial translational elongation
GFM2	G elongation factor, mitochondrial 2	mitochonrian	mitochondrial translation; ribosome disassembly
GFPT1	glutamine--fructose-6-phosphate transaminase 1	cytoplasm	carbohydrate biosynthetic process
GLMN	glomulin, FKBP associated protein	intracellular	positive regulation of cytokine secretion
GLO1	glyoxalase I	cytoplasm	carbohydrate metabolic process
GLUD1	glutamate dehydrogenase 1	cytoplasm; mitochondrial matrix	oxidation reduction
GNB2L1	guanine nucleotide-binding protein beta subunit 2-like 1	cytoplasm; cytoskeleton; cytosol; dendrite; midbody; mitochondrion; neuronal cell body; nucleus; perikaryon	SH2 domain binding
GPAM	glycerol-3-phosphate acyltransferase, mitochondrial	cytoplasm; mitochondrion outer membrane	acyl-CoA metabolic process
GPC2	glypican 2	cell membrane; endoplasmic reticulum; extracellular space	neuron differentiation

GPR98	G-protein coupled receptor 98	cytoplasm; membrane; secreted	G-protein coupled receptor protein signaling pathway
GPX4	glutathione peroxidase 4 (phospholipid hydroperoxidase)	mitochondrion; cytoplasm; nucleus	chromatin organization; spermatogenesis
GRPEL1	GrpE-like 1, mitochondrial	mitochondrial matrix	protein folding; protein import into mitochondrial matrix
GRPEL2	GrpE-like 2, mitochondrial	mitochondrial matrix	protein folding; protein import into mitochondrial matrix
GSN	gelsolin	actin; cytoplasm; cytoskeleton; extracellular space	apoptosis; vesicle-mediated transport
GST01	glutathione S-transferase omega 1	cytoplasm	L-ascorbic acid biosynthetic process
GTF2B	general transcription factor IIB	nucleus	RNA elongation from RNA polymerase II promoter/initiation/DNA-dependent
GTF2I	general transcription factor IIi	cytoplasm; nucleus	regulation of transcription; signal transduction
GTF2IRD1	GTF2I repeat domain containing 1	nucleus	regulation of transcription, DNA-dependent
GTPBP4	GTP binding protein 4	cytoplasm; nucleus	regulation of cell migration/ adhesion/collegen binding
GUK1	guanylate kinase 1	not well defined	purine nucleotide metabolic process
GYS1	glycogen synthase 1 (muscle)	cytoplasm; cytosol; inclusion body; membrane	glucose binding; glycogen synthase activity; protein binding
HAGH	hydroxyacylglutathione hydrolase	cytoplasm; mitochondrion	carbohydrate metabolic process; glutathione biosynthetic process
HARS	histidyl-tRNA synthetase	cytoplasm	histidyl-tRNA aminoacylation; translation
HAT1	histone acetyltransferase 1	cytoplasm; nucleus	chromatin modification; histone acetylation
HCN2	hyperpolarization activated cyclic nucleotide-gated potassium channel 2	membrane; synapse	cation transport; cell-cell signaling; response to hormone stiumulus; transmembrane transport
HDAC1	histone deacetylase 2	nucleus; cytoplasm	anti-apoptosis; protein amino acid deacetylation

HDAC2	histone deacetylase 2	cytoplasm; nucleus	cellular response to heat; chromatin remodeling; histone deacetylation
HDAC6	histone deacetylase 6	cytoplasm; nucleus	aggresome assembly; cellular response to hydrogen peroxide
HDAC7	histone deacetylase 7	cytoplasm; nucleus	B cell activation; B cell differentiation; cell-cell junction assembly
HDGF	HDGF hepatoma-derived growth factor	cytoplasm; extracellular space; nucleuss	signal transduction; transcription
HDLBP	high density lipoprotein binding protein	cytoplasm; nucleus; plasma membrane	cholesterol metabolic process; lipid transport; steroid metabolic process
HIF1AN	hypoxia inducible factor 1, alpha subunit inhibitor	nucleus	oxidation reduction; regulation of transcription
HINT1	histidine triad nucleotide binding protein 1	cytoplasm; nucleus	signal transduction
HIP1R	huntingtin interacting protein 1 related	cell/plasma membrane	regulation of endocytosis or receptor mediated
HIST3H2A	histone cluster 3, H2a	chromatin; nucleus	nucleosome assembly
HIST4H4	histone cluster 4, H4	chromatin; nucleus	nucleosome assembly
HK1	hexokinase 1	mitochondrion outer membrane; peripheral membrane	carbohydrate metabolic process; glycolysis
HK2	hexokinase 2	mitochondrion	carbohydrate metabolic process; glucose metabolic process
HMGA1	high mobility group AT-hook 1	cytoplasm; chromatin; nucleus	DNA replication; protein assembly; chromatin remodeling
HMGB1	high-mobility group box 1	cytoplasm; extracellular space; nucleus; plasma membrane	DNA ligation involved in DNA repair; DNA topological change; dendritic cell chemotaxis
HMGB2	High mobility group protein B2;High mobility group protein 2	condensed chromosome; cytoplasm; extracellular space; nucleoplasm, nucleuspeinuclear region of cytoplasm	DNA ligation involved in DNA repair; cell chemotaxis; chromatin organization
HMGB3	high-mobility group box 3	nucleus	DNA recombination
HMGB3	solute carrier family 9 (sodium/hydrogen exchanger), member 3 regulator 1	cytoplasm; plasma membrane	Wnt receptor signaling pathway

HNRNPD	heterogeneous nuclear ribonucleoprotein D	cytoplasm; nucleus; ribonucleosomes;	RNA splicing; RNA processing; mRNA stabilization
HNRNPM	heterogeneous nuclear ribonucleoprotein M	nucleus; plasma membrane	RNA splicing
HNRNPUL2	heterogeneous nuclear ribonucleoprotein U-like protein 2	nucleus	induction of cell death
HNRPLL	heterogeneous nuclear ribonucleoprotein L-like	nucleus; ribosome	RNA splicing; mRNA processing
HPRT1	hypoxanthine phosphoribosyltransferase 1	cytoplasm	hypoxanthine metabolic process; nucleoside metabolic process
HSD17B4	hydroxysteroid (17-beta) dehydrogenase 4	perioxosome	oxidation reduction; fatty acid metabolic process
HSDI7B12	hydroxysteroid (17-beta) dehydrogenase 12	endoplasmic membrane	oxidation reduction; positive regulation of cell-substrate adhesion
HSP90AA1	heat shock protein 90kDa alpha (cytosolic), class A member 1	cytoplasm	mitochondrial outer membrane translocase complex assembly
HSPA5	heat shock 70kDa protein 5 (glucose-regulated protein, 78kDa)	cell surface; pernuclear cytoplasm; ER-Golgi intermediate compartment; endoplasmic reticulum; melanosome; nucleus	ER-associated protein catabolic process; anti-apoptosis
IARS	isoleucyl-tRNA synthetase	cytoplasm	isoleucyl-tRNA aminoacylation
IDE	insulin-degrading enzyme	cytoplasm; mitochondrion; nucleus; peroxisome; extracellular space	proteolysis; signal transduction; beta-amyloid metabolic process
IDH3A	isocitrate dehydrogenase 3 (NAD+) alpha	mitochondrian	NADH metabolic process; carbohydrate metabolic process
IFRD1	interferon-related developmental regulator 1	nucleus	muscle cell differentiation; myoblast cell fate determination
IFT20	intraflagellar transport 20 homolog (Chlamydomonas)	centrosome; cytoplasm; golgi apparatus	cell projection organization
IGBP1	Immunoglobulin-binding protein 1	cytoplasm	B cell activation; regulation of signal transduction
IGF2R	cation-independent mannose-6-phosphate receptor	cytoplasm; endosome; extracellular space; membrane; lysosome membrane	positive regulation of apoptosis; response to retinoic acid
IKBKE	inhibitor of kappa light polypeptide gene enhancer in B-cells, kinase epsilon	cytoplasm; nucleus; endosome	DNA damage response, signal transduction resulting in induction of apoptosis; immune response

IL12RB2	interleukin 12 receptor, beta 2	cell/plasma membrane	cell surface receptor linked signaling pathway; peptidyl-tyrosine phosphorylation
ILF3	interleukin enhancer binding factor 3	cytoplasm; mitochondria; nucleolus; nucleus	positive or negative regulatin of transcription
IP6K2	inositol hexakisphosphate kinase 2	nucleus	negative regulation of cell growth; positive regulation of apoptosis; phosphoinositide phosphorylation
IRS1	Insulin receptor substrate-1	nucleus; caveola; cytoplasm; microsome; plasma membrane	cellular response to insulin stimulus; negative insulin receptor signaling pathway
ITGA3	integrin, alpha 3 (antigen CD49C, alpha 3 subunit of VLA-3 receptor)	Membrane; Single-pass type I membrane protein; synaptosome	cell-matrix adhesion; neuron migration; integrin-mediated signaling pathway
ITGAL	integrin, alpha L (antigen CD11A (p180), lymphocyte function-associated antigen 1	membrane	heterophilic cell-cell adhesion; inflammatory response; leukocyte cell-cell adhesion; signal transduction
ITGAV	integrin, alpha V (vitronectin receptor, alpha polypeptide, antigen CD51)	plasma membrane	apoptotic cell clearance; cell adhesion
ITIH1	inter-alpha (globulin) inhibitor H1	secreted/extracellular	hyaluronan metabolic process; leukocyte activation
ITPR2	inositol 1,4,5-triphosphate receptor, type 2	cytoplasm; integral to membrane; microsome; sarcoplasmic reticulum membrane;	calcium ion transport; ion transport/transmembrane; signal transduction
ITSN1	intersectin 1 (SH3 domain containing)	cell junction; cell projection; cytoplasm; endomembrane system; lamellipodium; synapse	apoptosis; regulation of Rho protein signal transduction
IVD	Isovaleryl-CoA dehydrogenase	mitochondrial matrix	leucine catabolic process; oxidation reduction
JPH2	junctophilin 2	endoplasmic reticulum membrane; plasma membrane; sarcoplasmic reticulum membrane	calcium ion transport into cytosol; regulation of ryanodine-sensitive calcium-release channel activity
JPH3	junctophilin3	cell/plasma membrane; endoplasmic reticulum; microsome; sarcoplasmic reticulum	calcium homeostsis; cell migration/cell-cell migration/collagen
JUN	jun proto-oncogene	nucleus	response to cytokine stimulus; angiogenesis; cellular response to potassium ion starvation
KARS	lysyl-tRNA synthetase	cytoplasm; membrane; mitochondrion; nucleus	lysyl-tRNA aminoacylation; tRNA processing
KAT2A	K(lysine) acetyltransferase 2A	nucleus	cell proliferation; chromatin remodeling; histone deubiquitination
KCNG3	potassium voltage-gated channel, subfamily G, member 3	cytoplasm; endoplasmic reticulum; membrane	transmembrane transport; ion transport

KCNQ1	potassium voltage-gated channel, KQT-like subfamily, member 1	Membrane; Multi-pass membrane protein	gene silencing; ion transport
KCNQ2	potassium voltage-gated channel, KQT-like subfamily, member 2	Membrane; Multi-pass membrane protein.	potassium ion transport; synaptic transmission; transmembrane transport
KIF13A	kinesin family member 13A	cytoplasm/microtubule	regulation of microtubule-based movement; protein transport
KIF1B	kinesin family member 1B	cytoplasm; cytoskeleton; mitochondria	anterograde axon cargo transport; apoptosis; cytoskeleton-dependent intracellular transport
KIF2C	kinesin family member 2C	cytoplasm/microtubule; Nucleus; chromatin	cell cycle; cell division; cell proliferation; microtubule depolymerization
KIF3B	kinesin family member 3B	cytosol/microtubule	microtubule based movement
KIFC1	kinesin family member C1	centrosome; early endosome; nucleus	cell cycle; mitotic sister chromatid segregation
KLC1	kinesin light chain 1	cytosol; microtubule	microtubule based movement
KLF2	kruppel-like factor 2	nucleus	metal ion binding; protein binding
KPNA4	karyopherin alpha 4 (importin alpha 3)	cytoplasm; nucleus	intracellular protein transport
LAMC1	laminin, gamma 1 (formerly LAMB2)	Secreted › extracellular space › extracellular matrix › basement membrane.	cell adhesion; cell migration; extracellular matrix disassembly
LARS	leucyl-tRNA synthetase	cytoplasm	leucyl-tRNA aminoacylation
LASS2	LAG1 homolog, ceramide synthase 2	endoplasmic reticulum membrane; nuclear membrane	lipid biosynthesis; sphingolipid metabolic process; transcription
LDLRAP1	low density lipoprotein receptor adaptor protein 1	cytoplasm; cell/plasma membrane	cholesterol homeostasis/metabolism/transport/steroid metabolism
LEO1	Leo1, Paf1/RNA polymerase II complex component, homolog	nucleus	transcription
LGALS3	lectin, galactoside-binding, soluble, 3	cytoplasm; cell/plasma membrane; extracellular proteinacious space; mitochondrion; nucleus	cell differentiation; extracellular matrix organization
LIG1	ligase I, DNA, ATP-dependent	nucleus	DNA recombination; DNA repair; DNA replication; Cell cycle;

LIN7C	lin-7 homolog C	cell junction; membrane	exocytosis; neurotransmitter secretion
LRPAP1	alpha-2-macroglobulin receptor-associated protein	cell surface; cytoplasm; endoplasmic reticulum	cell proliferation; vesicle-mediated transport
LSM3	LSM3 homolog, U6 small nuclear RNA associated	nucleus	mRNA processing; RNA splicing
LSM5	LSM5 homolog, U6 small nuclear RNA associated	nucleus	RNA splicing
LTA4H	leukotriene A4 hydrolase	cytoplasm; nucleus	inflammatory response; proteolysis
MACF1	microtubule-actin crosslinking factor 1	cytoplasm; cytoskeleton	Wnt receptor signaling pathway;
MAD1L1	MAD1 mitotic arrest deficient-like 1	centrosome; cytoplasm; kinetochore; nucleus	cell cycle; cell division
MAN2A1	mannosidase, alpha, class 2A, member 1	golgi; membrane	N-glycan processing; carbohydrate metabolic process
MAP1B	microtubule-associated protein 1B	cytoplasm; cell/plasma membrane	induction of synaptic plasticity by chemical substance
MAP1LC3A	microtubule-associated protein 1 light chain 3 alpha	cytoplasm; endomembrane system; autophagosome membrane	autophagic vacuole assembly
MAP2K2	mitogen-activated protein kinase kinase 2	cytosol	MAPKKK cascade; Ras protein signal transduction
MAP2K4	mitogen-activated protein kinase kinase 4	not well defined	protein amino acid phosphorylation; signal transduction; JNK cascade
MAP2K7	mitogen-activated protein kinase kinase 7	cytoplasm; nucleus	protein amino acid phosphorylation
MAP3K3	mitogen-activated protein kinase kinase kinase 3	cytoplasm	MAPKKK cascade
MAPK1	mitogen-activated protein kinase 1	cytoplasm; nucelus	cell cycle; chemotaxis; positive regulation of transcription
MARS2	methionyl-tRNA synthetase 2, mitochondrial	mitochondrial matrix; cytoplasm	methionyl-tRNA aminoacylation
MBP	myelin basic protein	plasma membrane	immune response; myelination; negative regulation of axonogenesis; synaptic transmission

MCM2	minichromosome maintenance complex component 2	chromatin; nucleus	DNA replication; DNA unwinding involved in replication
MCM3	minichromosome maintenance complex component 3	cytoplasm; nucelus	DNA replication; cell cycle
MCM4	minichromosome maintenance complex component 4	nucleus	DNA replication; regulation of transcription; DNA unwinding involved in replication
MCM5	minichromosome maintenance complex component 5	nucelus	DNA replication; regulation of transcription
MCM6	minichromosome maintenance complex component 6	nucelus	regulation of transcription; cell cycle; DNA replication
MDH1	malate dehydrogenase 1, NAD	cytoplasm; mitochondrion	cellular carbohydrate metabolic process
ME2	malic enzyme 2, NAD(+)-dependent, mitochondrial	mitochondrial matrix	oxidation reduction; malate metabolic process
MEA1	male-enhanced antigen 1	cytoplasm	cell differentiation; spermatogenesis
MECP2	methyl CpG binding protein 2	nucleus	negative regulation of transcription
MED13	mediator complex subunit 13	nucleus	androgen receptor signaling pathway
METAP1	methionine aminopeptidase 1	cytoplasm	proteolysis; regulation of translation
METTL3	methyltransferase like 3	nucleus speckle	RNA methylation; nucleobase, nucleoside, nucleotide and nucleic acid metabolic process
MGST1	microsomal glutathione S-transferase 1	Mitochondrion membrane; endoplasmic reticulum; microsome; nucleus; peroxisomal membrane	leydig cell differentiation; glutathione metabolic process
MKI67	antigen identified by monoclonal antibody Ki-67	nucleus; cytoplasm	cell cycle; meiosis
MKNK2	MAP kinase interacting serine/threonine kinase 2	not well defined	protein amino acid phosphorylation; regulation of translation
MLLT3	myeloid/lymphoid or mixed-lineage leukemia (trithorax homolog, Drosophila); translocated to, 3	nucleus; cytoplasm	regulation of transcription, DNA-dependent
MMP13	matrix metallopeptidase 13 (collagenase 3)	extracellular space ; extracellular matrix	collagen catabolic process; proteolysis

MNT	MAX binding protein	nucleus	regulation of cell cycle
MOCS1	molybdenum cofactor synthesis 1	nucleus	mo-molybdopterin cofactor biosynthetic process
MPHOSPH10	M-phase phosphoprotein 10 (U3 small nucleolar ribonucleoprotein)	nucleolus; nucleus	mRNA processing ; rRNA processing
MPND	MPN domain-containing protein	undefined	peptidase
MRPS28	mitochondrial ribosomal protein S28	mitochondrian; mitochondrial small ribosomal subunit	poly (A) RNA binding
MSH6	mutS homolog 6	nucleus; chromatin	DNA damage response
MTA1	metastasis associated 1	nucleus; cytoplasm	regulation of transcription, DNA-dependent; signal transduction
MTA2	metastasis associated 1 family, member 2	nucleus	chromatin assembly or disassembly
MTA3	metastasis associated 1 family, member 3	cytoplasm; nucleus	regulation of transcription, DNA-dependent
MTAP	methylthioadenosine phosphorylase	cytoplasm	nucleobase, nucleoside, nucleotide and nucleic acid metabolic process
MTHFD1	methylenetetrahydrofolate dehydrogenase (NADP+ dependent) 1, methenyltetrahydrofolate cyclohydrolase, formyltetrahydrofolate synthetase	cytoplasm; mitochondrion	oxidation reduction; folic acid and derivative biosynthetic proces
MTMR3	myotubularin related protein 3	cytoplasm; cell/plasma membrane	dephosphorylation
MTTP	microsomal triglyceride transfer protein	endoplasmic reticulum	lipid metabolic process
MTX1	metaxin 1	mitochondrion outer membrane	protein transport
MVP	major vault protein	cytoplasm; nucleus	mRNA transport; protein transport; transmembrane transport
MYBBP1AN	MYB binding protein (P160) 1a	cytoplasm; nucleolus; nucleus	protein transport; transcription
MYCBP	c-myc binding protein	centrosome; cytoskeleton; cytoplasm; kinetochore; microtubule; mitochondrion; nucleus	regulation of transcription; spermatogenesis

MyD88	myeloid differentiation primary response protein 88	cytoplasm; intrinsic to membrane	I-kappaB kinase/NF-kappaB cascade; anti-apoptosis
Myh 10	myosin, heavy polypeptide 10, non-muscle	cytoplasm	exocytosis; myofibril assembly; plasma membrane repair; axonogenesis
MYH10	myosin, heavy chain 10, non-muscle	cytoplasm; myosin	actin filament-based movement; cytokinesis after mitosis
MYH9	myosin, heavy chain 9, non-muscle	cytoplasm; cell/plasma membrane; myosin or actin; nucleus	actin cytoskeleton reorganization; angiogenesis; regulation of cell shape
MYO18A	myosin XVIIIA	cytoplasm; golgi apparatus;ER-golgi intermediate compartment; myosin complex; nucleolus; nucleus	anti-apoptosis
MYO1C	myosin IC	cytoplasm; membrane; nucleus	mRNA transport; protein transport; transmembrane transport
NACA	nascent polypeptide-associated complex alpha subunit	cytoplasm; nucleus	translation; transcription
NADSYN1	NAD synthetase 1	cytoplasm; cytosol	NAD biosynthetic process;NAD metabolic process; small molecule metabolic process
NAGA	N-acetylgalactosaminidase, alpha-	lysosome	carbohydrate catabolic process; glycolipid catabolic process
NAP1L1	nucleosome assembly protein 1-like 1	melansome; nucleus	DNA replication; nucleosome assembly; positive regulation of cell proliferation
NCAPH	non-SMC condensin I complex, subunit H	chromatin; cytoplasm; nucleus	cell division; mitosis; mitotic cell cycle; mitotic chromosome condensation
NCKAP1L	NCK-associated protein 1-like	SCAR complex; cytoplasm; extracellular exosome; membrane	rac GTPase activator; protein binding
NCL	nucleolin	cytoplams; membrane; nucleolus; nucleus	ribosomal biogensis; glucocorticoid receptor signaling pathway
NDUFA7	NADH dehydrogenase (ubiquinone) 1 alpha subcomplex, 7, 14.5kDa	mitochondrion inner membrane; peripheral membrane protein	mitochondrial electron transport, NADH to ubiquinone transport
NDUFAB	NADH dehydrogenase (ubiquinone) 1 alpha subcomplex, 8, 19kDa	mitochondrion	NADH dehydrogenase activity and oxidoreductase activity
NDUFS3	NADH dehydrogenase (ubiquinone) Fe-S protein 3, 30kDa (NADH-coenzyme Q reductase)	mitochondrion	electron transport chain; induction of apoptosis
NDUFS4	NADH dehydrogenase (ubiquinone) Fe-S protein 4, 18kDa (NADH-coenzyme Q reductase)	mitochondrian	electron transport chain

NEDD4	neural precursor cell expressed, developmentally down-regulated 4	cytoplasm	cellular response to UV
NFKB1	nuclear factor of kappa light polypeptide gene enhancer in B-cells 1	Nucleus. Cytoplasm. Nuclear, but also found in the cytoplasm in an inactive form complexed to an inhibitor (I-kappa-B)	anti-apoptosis
NIF3L1	NIF3 NGG1 interacting factor 3-like 1	cytoplasm; mitochondrion	positive regulation of transcription, DNA-dependent
NME2	non-metastatic cells 2, protein (NM23B) expressed in	centrosome; cytoplasm; cytosol; vesciular exosome; lamellipodium; membrane; nucleus; perinuclear region of cytoplasm; ruffle	ATP binding; RNA polymerase II regulator regions DNA binding; gamma-tubulin binding
NMT1	N-myristoyltransferase 1	cytoplasm	N-terminal protein lipidation; N-terminal protein myristoylation; protein lipoylation
NOC2L	nucleolar complex associated 2 homolog	nucleus, nucleolus	chromatin binding; poly(A) RNA bidnign; transcription repression;
NOS3	nitric oxide synthase 3 (endothelial cell)	caveola; golgi apparatus; cytoplasm/cytoskeletonh; nucleus/nucleolus; plasma membrane	anti-apoptosis; arginine catabolic process
NPM1	nucleophosmin (nucleolar phosphoprotein B23, numatrin)	cytoplasm; nucleus; ribosome	cell growth/proliferation; protein transport
NSF	N-ethylmaleimide-sensitive factor	cytoplasm; dendritic shaft	proteolysis; regulation of exocytosis
NUCKS	nuclear casein kinase and cyclin-dependent kinase substrate 1	cytoplasm; nucleus	Poly (A) RNA binding
NUDT2	nudix (nucleoside diphosphate linked moiety X)-type motif 2	mitochondrion	induction of apoptosis; nucleobase, nucleoside, nucleotide and nucleic acid metabolic process
NUP133	nucleoporin 133kDa	kinetochore; nuclear pore complex;	protein transport; transmembrane transport; nuclear pore organization
OPA1	optic atrophy 1 (autosomal dominant)	mitochondrion	apoptosis
OXSR1	oxidative-stress responsive 1	undefined	phosphorylation; response to oxidative stress
PAFAH1B1	platelet-activating factor acetylhydrolase 1b, regulatory subunit 1	centrosome; cytoplasm; cytoskeleton; microtubules; nucleus	Cell cycle; cell differentiation; cytoskeleton organization
PAFAH1B3	platelet-activating factor acetylhydrolase 1b, catalytic subunit 3	cytoplasm	spermatogenesis; lipid catabolic process
PAPOLA	poly(A) polymerase alpha	cytoplasm; nucleus	RNA splicing; mRNA processing; mRNA polyadenylation

PAPSS2	3'-phosphoadenosine 5'-phosphosulfate synthase 2	undefined	adenylylsulfate kinase activity; nucleotide bidning;
PARD6A	par-6 partitioning defective 6 homolog alpha	cytoplasm; cell/plasma membrane; nucleus	cell cycle; cell division; viral reproduction
PARP1	poly [ADP-ribose] polymerase-1	nucleus	protein amino acid ADP-ribosylation; protein amino acid ADP-ribosylation; apoptosis
PASK	PAS domain containing serine/threonine kinase	cytoplasm; Golgi apparatus	regulation of transcription, DNA-dependent; protein amino acid phosphorylation
PC	pyruvate carboxylase	cytoplasm; mitochondrial matrix	oxaloacetate metabolic process; lipid biosynthetic process
PCDH11Y	protocadherin 11 Y-linked	membrane	homophilic cell adhesion
PCYT1A	phosphate cytidylyltransferase 1, choline, alpha	cytoplasm; membrane	phospholipid biosynthetic process
PDCD6IP	programmed cell death 6 interacting protein	centrosome; cytoplasm; melanosome.	apoptosis; cell cycle; cell division; protein transport
PDE1B	phosphodiesterase 1B, calmodulin-dependent	cytoplasm	apoptosis; signal transduction; cAMP catabolic process; cGMP catabolic process; visual learning
PDIA4	protein disulfide isomerase family A, member 4	endoplasmic reticulum; melansome	cell redox homeostasis; protein secretion
PDIA6	protein disulfide isomerase family A, member 6	endoplasmic reticulum; golgi apparatus cell/plasma membrane; melanosome;	cell redox homeostasis; protein folding
PDK1	pyruvate dehydrogenase kinase, isozyme 1	mitochondrial matrix	carbohydrate metabolic process; glucose metabolic process
PDLIM1	PDZ and LIM domain 1	cytoplasm; cytoskeleton; actin	regulation of transcription; response to hypoxia; response to oxidative stress
PDLIM2	PDZ and LIM domain 2 (mystique)	cytoplasm; cytoskeleton; myosin/actin; nucleus	suppresses Anchorage-independent growth; cell migration/cell-cell migration/collagen
PEPD	peptidase D	not well defined	peptidase activity; proteolysis
PEX6	peroxisomal biogenesis factor 6	cytoplasm; peroxisome membrane	peroxisome organization; protein import into peroxisome matrix, translocation
PGK1	phosphoglycerate kinase 1	cytoplasm	glycolysis; phosphorylation

PGM1	phosphoglucomutase 1	cytoplasm	carbohydrate metabolic process; spermatogenesis
PGR	Progesterone receptor	cytoplasm; intracellular membrane; nucleus	transcription (negative); cell proliferation; signal transduction
PGRMC1	progesterone receptor membrane component 1	endoplasmic reticulum; integral membrane component; nucleolus; vesciular exosome	heme binding; protein bindign; steroid binding;
PHACTR3	phosphatase and actin regulator 3	nuclear matrix-intermediate filament scaffold	phosphatase inhibitor
PIK3C2G	phosphoinositide-3-kinase, class 2, gamma polypeptide	membrane	phosphoinositide-mediated signaling
PIK3R4	phosphoinositide-3-kinase, regulatory subunit 4	cytoplasm	phosphorylation
PKD2	polycystic kidney disease 2 (autosomal dominant)	endoplasmic reticulum; membrane	JAK-STAT cascade
PKD2	polycystic kidney disease 2	actin cytoskeleton; endoplasmic reticulum; plasma membrane	JAK-STAT cascade; calcium ion transport; cation transport
PKP3	plakophilin 3	cell junction; desmosome; nucleus	cell adhesion
PLD3	phospholipase D family, member 3	cytoplasm; endoplasmic reticulum membrane; golgi apparatus membrane; lipid-anchor	lipid catabolic process
PLDN	pallidin homolog	cytoplasm; endomembrane system; endosome; peripheral membrane protein	post-Golgi vesicle-mediated transport; exocytosis
PLEC	plectin	cytoskeleton; cytoplams; plasma membrane	hemidesmosome assembly
PLEK	pleckstrin	cytoplasm; extracellular region	actin cytoskeleton reorganization; hemopoietic progenitor cell differentiatioN
PLOD1	procollagen-lysine 1, 2-oxoglutarate 5-dioxygenase 1	rough endoplasmic reticulum membrane	Oxidation reduction; response to hypoxia; hydroxylysine biosynthetic process
PLOD3	procollagen-lysine, 2-oxoglutarate 5-dioxygenase 3	rough endoplasmic reticulum membrane	cellular response to hormone stimulus; Oxidation reduction
PMM2	phosphomannomutase 2	cytoplasm	protein amino acid glycosylation; GDP-mannose biosynthetic process
PMVK	phosphomevalonate kinase	peroxisome; cytoplasm	cholesterol biosynthetic process; lipid metabolic process; protein amino acid phosphorylation

POFUT2	protein O-fucosyltransferase 2	endoplasmic reticulum	carbohydrate metabolic process; fucose metabolic process
POLA1	polymerase (DNA directed), alpha 1, catalytic subunit	colocalizes with chromatin; cytoplasm; nucleus	NOT DNA repair; DNA replication
POLD1	polymerase (DNA directed), delta 1, catalytic subunit 125kDa	nucelus	DNA repair; DNA replication
POLD3	polymerase (DNA-directed), delta 3, accessory subunit	nucleus	DNA synthesis involved in DNA repair
POLDIP3	polymerase (DNA-directed), delta interacting protein 3	cytoplasm; nucleus	translation
POLR1C	polymerase (RNA) I polypeptide C	nucleus	rRNA processing/transcription
POLR2A	polymerase (RNA) II (DNA directed) polypeptide A, 220kDa	nucleolus; nucleus	RNA splicing; transcription
PPA1	pyrophosphatase (inorganic) 1	cytoplasm	phosphate metabolic process; diphosphate metabolic process
PPA2	pyrophosphatase (inorganic) 2	mitochondrian; cytoplasm	diphosphate metabolic process
PPIG	peptidylprolyl isomerase G	nucleus matrix	RNA splicing
PPME1	protein phosphatase methylesterase 1	not well defined	protein amino acid demethylation
PPP1R14A	protein phosphatase 1, regulatory (inhibitor) subunit 14A	cytoplasm	phosphatase inhibitor
PPP2CA	protein phosphatase 2, catalytic subunit, alpha isozyme	cytoplasm; plasma membrane; mitochondrion; nucleus	RNA splicing; apoptosis; meiosis
PPP2R2B	protein phosphatase 2, regulatory subunit B, beta	cytoplasm; mitochondrion	apoptosis; cell death; signal transduction
PPP2R5E	protein phosphatase 2, regulatory subunit B', epsilon isoform	cytoplasm; intracellular membrane	signal transduction
PPP5C	protein phosphatase 5, catalytic subunit	cytoplasm; nucleus	mitosis
PRDX1	peroxiredoxin 1	cytoplasm; melanosome; mitochondrion; nucleus	cell proliferation

PRDX4	peroxiredoxin 4	cytoplasm; extracellular space; mitochondrion	I-kappaB phosphorylation; cell redox homeostasis
PRDX6	peroxiredoxin 6	cytoplasm; lysosome; nucleus	cell redox homeostasis
PRIM1	primase, DNA, polypeptide 1	nucleoplasm	DNA replication; transcription
PRKACA	protein kinase, cAMP-dependent, catalytic, alpha	cytoplasm; cell/plasma membrane; mitochondrion; nucleus	glutamatergic synaptic transmision; atp synthesis; cell cycle; phosphorylation
PRKD3	protein kinase D3	cytoplasm; membrane	phosphorylation
PRKG2	protein kinase, cGMP-dependent, type II and I	cytoplasm	protein amino acid phosphorylation; signal transduction
PRMT3	protein arginine methyltransferase 3	cytoplasm; ribosome	histone-arginine N-methytransferase;
PRPF3	PRP3 pre-mRNA processing factor 3 homolog	nucleus speckle	RNA splicing; mRNA processing; visual perception
PRPF40A	PRP40 pre-mRNA processing factor 40 homolog A	chromatin; nucleus	mRNA processing
PRPSAP1	phosphoribosyl pyrophosphate synthetase-associated protein 1	cytoplasm	nucleobase, nucleoside, nucleotide and nucleic acid metabolic process
PRPSAP2	phosphoribosyl pyrophosphate synthetase-associated protein 2	cytoplasm	nucleobase, nucleoside, nucleotide and nucleic acid metabolic process
PSIP1	PC4 and SFRS1 interacting protein 1	cytoplasm; nucleus	stress-induced apoptosis
PSMA6	proteasome (prosome, macropain) subunit, alpha type, 6	cytoplasm; nucleus; mitochondrion	ubiquitin-dependent protein catabolic process; cell cycle; proteolysis
PSMB3	proteasome (prosome, macropain) subunit, beta type, 3	cytoplasm; nucleus	ubiquitin-dependent protein catabolic process; proteolysis; cell cycle
PTGES3	prostaglandin E synthase 3 (cytosolic)	cytoplasm	glucocorticoid receptor signaling pathway; glycogen biosynthetic process
PTGFR	prostaglandin F receptor (FP)	extracellular space; membrane	G-protein coupled receptor protein signaling pathway
PTPN11	protein tyrosine phosphatase, non-receptor type 11	cytoplasm; mitochondrion	signal transduction; activation of MAPK activity; axonogenesis; glucose homeostasis

PTPN12	protein tyrosine phosphatase, non-receptor type 12	cytoplasm	dephosphorylation
PTPN12	PTPN12 protein tyrosine phosphatase, non-receptor type 12	cytoplasm	phosphatase
PTRF	polymerase I and transcript release factor	caveola; endoplasmic reticulum; mitochondrion	caveolae formation and stabilization; lipid metabolism
RAB11FIP5	RAB11 family interacting protein 5 (class I)	cytoplasm; endosome membrane; mitochondion membrane	transport
RABEP1	rabaptin, RAB GTPase binding effector protein 1	cytoplasm; early endosome	apoptosis; endocytosis; protein transport; cellular membrane fusion
RABGAP1	RAB GTPase activating protein 1	cytoplasm	regulation of Rab GTPase activity; cell cycle
RABIF	RAB interacting factor	cytoplasm	cellular membrane fusion; protein transport; small GTPase mediated signal transduction
RALBP1	ralA binding protein 1	membrane	signal transduction; small GTPase mediated signal transduction
RAN	RAN, member RAS oncogene family	cytoplasm; melanosome; nucleus	cell cycle; cell division; mitosis; protein transport
RANBP1	RAN binding protein 1	cytoplasm; nucleus	signal transduction
RAP1B	RAP1B, member of RAS oncogene family	cytosol; plasma membrane	signal transduction; Cell proliferation; small GTPase mediated signal transduction
RAPGEF2	Rap guanine nucleotide exchange factor (GEF) 2	synaptic plasma membrane	MAPKKK cascade; cAMP-mediated signaling
RARS	arginyl-tRNA synthetase	cytoplasm; nucleus; mitochondrion	aspartyl-tRNA aminoacylation
RASA2	RAS p21 protein activator 2	cell membrane; cytoplasm	intracellular signaling pathway; small GTPase mediated signal transduction
RASA3	RAS p21 protein activator 3	plasma membrane	signal transduction; small GTPase mediated signal transduction
RBBP7	retinoblastoma binding protein 7	nucleus	DNA replication; transcription; chromatin remodeling; cell growth
RBM28	RNA binding motif protein 28	nucleus	rRNA processing; RNA splicing

RBM28	RNA binding motif protein 28	nucleolus; nucleus	mRNA processing
RBM39	RNA binding motif protein 39	nucleus	transcription
RENBP	renin binding protein	not well defined	regulation of blood pressure; mannose metabolic process; N-acetylglucosamine metabolic process
RGS9	regulator of G-protein signaling 9	cytoplasm; membrane; nucleus	negative regulation of signal transduction
RNPEP	arginyl aminopeptidase (aminopeptidase B)	extracellular space; plasma membrane	proteolysis; leukotriene biosynthetic process
RPLP0	ribosomal protein, large, P0	cytoplasm; membrane; nucleus; ribonucleoprotein complex	RNA binding; translation
RPLP2	ribosomal protein, large, P2	cytoplasm; membrane; nucleus; ribonucleoprotein complex	RNA binding; translation
RPN1	ribophorin I	endoplasmic membrane; melansome	protein amino acid glycosylation
RPS6KA2	ribosomal protein S6 kinase, 90kDa, polypeptide 2	cytoplasm; nucleus	signal transduction; protein amino acid phosphorylation
RPS6KA3	ribosomal protein S6 kinase, 90kDa, polypeptide 3	cytoplasm; nucleus	protein amino acid phosphorylation
RRAS2	related RAS viral (r-ras) oncogene homolog 2	endoplasmic reticulum; membrane	signal transduction; cell migration
RRBP1	ribosome binding protein 1 homolog 180kDa	ribosome; endoplasmic reticulum; ribosome	Protein transport; signal transduction; translation; transmembrane transport
RRP9	ribosomal RNA processing 9, small subunit (SSU) processome component, homolog (yeast)	nucleus	rRNA processing
RTCD1	RNA terminal phosphate cyclase domain 1	nucleoplasm; nucleus	regulation of mRNA processing; axonogenesis
RTF1	Rtf1, Paf1/RNA polymerase II complex component, homolog	cytoplasm; nucleolus; nucleus	transcription
RTN4	reticulon 4	cytoplasm; endoplasmic reticulum membrane	negative regulation of anti-apoptosis; cell differentiation; angiogenesis
SARNP	SAP domain containing ribonucleoprotein	nucleus	regulation of translation

SCFD1	sec1 family domain containing 1	cytoplasm; endoplasmic reticulum; Gogli apparatus; plasma membrane	protein transport; post-Golgi vesicle-mediated transport; vesicle mediated transport
SDC2	syndecan 2	synapse; cell/plasma membrane	wound healing; response to hypoxia
SDPR	serum deprivation response protein	cytoplasm; caveola; membrane	cellular response to starvation
SEC23IP	SEC23 interacting protein	cytoplasm; endoplasmic reticulum	protein transport; Golgi organization
SEC63	SEC63 homolog	endoplasmic reticulum; cell/plasma membrane	protein localization at cell surface; protein transport/localization; protein refolding
SEPHS1	selenophosphate synthetase 1	cytoplasm; nucleus	selenophosphate synthesis
SEPT11	septin 11	cytoplasm	cell cycle; protein heterooligomerization; cell division
SEPT6	septin 6	cytoplasm	cell cycle; cytokinesis
SERPINE1	serpin peptidase inhibitor, clade E (nexin, plasminogen activator inhibitor type 1), member 1	cytoplasm; plasma membrane; secreted	cell proliferation; Pos. regulation of moncyte chemotaxsis
SERPINF1	serpin peptidase inhibitor, clade F (alpha-2 antiplasmin, pigment epithelium derived factor), member 1	secreted, melanosome	cell proliferation; response to retinoic acid; response to glucocorticoid stimulus
SF3A3	splicing factor 3a, subunit 3, 60kDa	nucleus	mRNA processing
SF3B1	splicing factor 3b, subunit 1, 155kDa	nucleus	mRNA processing
SFRS12	splicing factor, arginine/serine-rich 12	nucleus	RNA splicing; mRNA processing
SFTPD	surfactant protein D	lysosome; secreted	innate immune response; macrophage chemotaxis
SLC12A3	solute carrier family 12 (sodium/chloride transporters), member 3	cytoplasm; plasma membrane	sodium ion transport
SLC16A1	solute carrier family 16, member 1 (monocarboxylic acid transporter 1)	mitochondrion; plasma membrane	transmembrane transport; mevalonate transport; monocarboxylic acid transport
SLC26A5	solute carrier family 26, member 5 (prestin)	membrane	regulation of cell shape; sensory perception of sound

SLC39A7	solute Carrier family 39, member 7	endoplasmic reticulum membrane; membrane	ion transport
SLITRK2	SLIT and NTRK-like family, member 2	cell membrane	potassium ion transport; transmembrane transport; carbohydrate metabolic process
SLK	STE20-like kinase	cytoplasm; plasma membrane	apoptosis; nucleotide-excision repair; protein amino acid phosphorylation
SMAD4	SMAD family member 4	cytoplasm; nucleus	negative regulation of cell death
SMARCE1	SWI/SNF related, matrix associated, actin dependent regulator of chromatin, subfamily e, member 1	nucleus	chromatin modification; negative regulation of transcription
SMC4	structural maintenance of chromosomes 4	chromatin; cytoplasm; nucleus	cell division; cell cycle
SMN1	survival of motor neuron 1, telomeric	cytoplasm; nucleus	mRNA processing
SMS	spermine synthase	cytoplasm	methionine metabolic process; polyamine metabolic process; spermine biosynthetic process
SMYD3	SET and MYND domain containing 3	cytoplasm; nucleus	chromatin modification
SNRNP70	small nuclear ribonucleoprotein 70kDa (	nucleus	RNA splicing; mRNA processing
SNRPD2	small nuclear ribonucleoprotein D2 polypeptide 16.5kDa	cytoplasm; nucleus	RNA splicing; mRNA processing
SNRPG	small nuclear ribonucleoprotein polypeptide G	cytoplasm; nucleus	RNA splicing
SNX9	sorting nexin-9	cytoplasm; microsome; cell/plasma membrane	cell-cell signaling; protein transport/localization
SON	SON DNA binding protein	nucleus	anti-apoptosis
SORBS3	sorbin and SH3 Domain Containing 3	cell junction; cytoplasm; cytoskeleton; nucleus	positive regulation of cytoskeleton organization ;positive regulation of MAPKKK cascade; cell adhesion
SPARC	secreted protein, acidic, cysteine-rich	basement membrane; extracellular matrix	cellular response to growth factor stimulus; ossification; regulation of cell proliferation
SPAST	spastin	nucleus; cytoplasm	cell cycle; cell differentiation; cell division ; cell death;

SPTBN1	spectrin, beta, non-erythrocytic 1	cytoplasm; cytoskeleton; membrane; myofibril; nucleus; sarcomere	SMAD Protein nuclear translocation; actin filament capping
SR140	U2-associated SR140 protein	undefined	RNA splicing
SRRM1	serine/arginine repetitive matrix protein 1	nucleus matrix; nucleus speckle	mRNA processing
SRSF10	serine/arginine-rich splicing factor 10	cytoplasm; nucleus	RNA splicing, via transesterification reactions; regulation of transcription
SSB	Sjogren syndrome antigen B (autoantigen La)	cytoplasm; nucleus	RNA processing; histone mRNA metabolic process
SSU72	SSU72 RNA polymerase II CTD phosphatase homolog	cytoplasm; nucleus	phosphatase inhibitor; mRNA processing
SSX2IP	synovial sarcoma, X breakpoint 2 interacting protein	cell junction; nucleus	cell adhesion
ST13	suppression of tumorigenicity 13 (colon carcinoma) (Hsp70 interacting protein)	cytoplasm	protein folding
STARD4	STAR-related lipid transfer (START) domain containing 4	not well defined	lipid transport
STK24	serine/threonine-protein kinase 24	cytoplasm; nucleosome	signal transduction; phosphorylation
STMN1	stathmin 1	cytoplasm	cell differentiation; signal transduction; response to virus
STRN3	striatin, calmodulin binding protein 3	cytoplasm; gogi apparatus; membrane	transcription; negative regulation of estrogen receptor signaling pathway
SUB1	SUB1 homolog (S. cerevisiae)	nucleus	regulation of transcription from RNA polymerase II promoter
SUCLG1	succinate-CoA ligase, alpha subunit	mitochondrion	acetyl-CoA metabolic process; tricarboxylic acid cycle
SUPT6H	suppressor of Ty 6 homolog protein	nucleus	nucleobase, nucleoside, nucleotide and nucleic acid metabolic process; transcription
SYNCRIP	synaptotagmin binding, cytoplasmic RNA interacting protein	cytoplasm; endoplasmic reticulum; microsomes; nucleus	RNA splicing; mRNA processing
SYNPO	synaptopodin	cytoplasm; cytoskeleto; cell junction; dendritic spine; membrane; perikaryon; synapse	positive regulation of actin filament bundle assembly

TARS	Threonyl-tRNA synthetase	cytoplasm; mitochondria	threonyl-tRNA aminoacylation; translation
TAS2R4	taste receptor, type 2, member 4	membrane	sensory perception of taste; signal transduction
TBC1D1	TBC1 (tre-2/USP6, BUB2, cdc16) domain family, member 1	nucleus	regulation of Rab GTPase activity
TBP	TATA box binding protein	cytoplasm; nucleus	spermatogenesis; cell death; RNA elongation; transcription from RNA polymerase III promoter/DNA dependent
TBPL1	TBP-like 1	cytoplasm; Nucleus	regulation of transcription, DNA-dependent; spermatid nucleus differentiation
TCEA1	transcription elongation factor A (SII), 1	nucleus	RNA elongation; transcription from RNA polymerase II promoter
TCERG1	transcription elongation regulator 1	nucleus	transcription; transcription from RNA polymerase II promoter
TCF7L2	transcription factor 7-like 2 (T-cell specific, HMG-box)	cytoplasm; nucleus	anti-apoptosis; canonical Wnt receptor signaling pathway; cell cycle arrest
TCOF1	treacher Collins-Franceschetti syndrome 1	nucleolus; nucleus	methylation; rRNA trasncription
TCP1	t-complex 1	cytoplasm	protein folding; tubulin complex assembly
TDP1	tyrosyl-DNA phosphodiesterase 1	cytoplasm; nucleus	DNA ligation or repair or damage response; induction of cell death
TFIP11	tuftelin interacting protein 11	cytoplasm; nucleus	RNA splicing; mRNA processing
TFRC	transferrin receptor	cytoplasm; melanosome; endosome; membrane; mitochondrion	response to retinoic acid; response to hypoxia; proteolysis; response to nutrient
THBS1	thrombospondin 1	extracellular space; plasma membrane	apoptosis; cell adhesion; cell cycle arrest; cell migration; negative regulation of angiogenesis
THOC4	THO complex 4	cytoplasm; nucleus	RNA splicing; mRNA processing
THRAP3	thyroid hormone receptor associated protein 3	intracellular membrane; mediator complex; nucleus	androgen receptor signaling pathway; steroid hormone receptor signaling pathway
TIMM8B	translocase of inner mitochondrial membrane 8 homolog B	mitochondrion inner membrane; peripheral membrane	protein targeting to mitochondrion; sensory perception of sound; transmembrane transport

TIMP4	TIMP metallopeptidase inhibitor 4	secreted	response to cytokine stimulus; response to lipopolysaccharide
TIPRL	TIP41, TOR signaling pathway regulator-like	cytoplasm	DNA damage checkpoint; negative regulation of protein phosphatase type 2A activity
TJP1	tight junction protein 1 (zona occludens 1)	cell junction; membrane; nucleus	blastocyst formation; cell-cell junction assembly
TLN1	talin 1	cytoplasm; plasma membrane	cell-cell junction assembly; cytoskeleton organization
TLR8	toll-like receptor 8	membrane	I-kappaB kinase/NF-kappaB cascade
TLR9	toll-like receptor 9	cytoplasm; endoplasmic reticulum; endosome; lysosome; membrane	I-kappaB kinase/NF-kappaB cascade (positive and negative); defense response to bacterium
TMPO	thymopoietin	nucleus	regulation of transcription
TMSB10	thymosin beta 10	cytoplasm; cytoskeleton	cytoskeleton organization
TNPO1	transportin 1	cytoplasm; nucleus	protein import into nucleus, translocation
TNPO3	transportin 3	cytoplasm; Nucleus	Protein transport
TOMM20	translocase of outer mitochondrial membrane 20 homolog	mitochondrion outer membrane	protein targeting to mitochondrion
TOP1	topoisomerase (DNA) I	cytoplasm; nuceloplasm; nucleus;	DNA replication; DNA topological change; phosphorylation; programmed cell death
TOP2b	topoisomerase (DNA) II beta	nucleolus; nucleus	DNA topological change; chromosome condensation/segregation
TOPA2	topoisomerase (DNA) II alpha 170kDa	cytoplasm; nucleus	DNA replication; DNA topological change
TP53	tumor protein p53	cytoplasm; endoplasmic reticulum; mitchondrion; nucelus	ER overload response; apoptosis; cell cycle arrest; cell differentiation
TPM1	tropomyosin 1 (alpha)	cytoplasm; cytoskeleton	cellular component movement; sarcomere organization
TPM2	tropomyosin 2 (beta)	cytoplasm; cytoskeleton	ATPase activity; muscle contraction

TRAF2IP3	TRAF3 interacting protein 3	membrane	positive regulation of JNK cascade; cell growth
TRIM16	tripartite motif-containing 16	cytoplasm	histone H3 acetylation; histone H4 acetylation
TRIM2	tripartite motif-containing 2	cytoplasm; early endosome	protein transport
TRIM3	tripartite motif-containing 3	cytoplasm; early endosome	protein transport
TRNT1	tRNA nucleotidyl transferase, CCA-adding, 1	mitochondrium	RNA processing; protein targeting to mitochondrion; tRNA 3'-end processing
TRPM6	transient receptor potential cation channel, subfamily M, member 6	membrane	calcium homeostsis; magnesium homeostatis/transport
TSFM	Ts translation elongation factor, mitochondrial	mitochonrian; nucleus	translational elongation
TTC4	chaperonin containing TCP1, subunit 6A (zeta 1)	cytoplasm	protein folding
TUBG1	tubulin, gamma 1	cytoplasm	meiotic spindle organization
TUBG1	tubulin, gamma 1	centrosome; cytoplasm	meiotic spindle organization; microtubule cytoskeleton organization; protein polymerization
TWF1	twinfilin, actin-binding protein, homolog 1	cytoplasm; cytoskeleton; ruffles	protein amino acid phosphorylation
TXLNA	taxilin alpha	cytoplasm; extracellular region	exocytosis; cell proliferation
TXNDC9	thioredoxin domain containing 9	centrosome; cytoplams; midbody; nucleus	biological process; cell redox homeostasis
TXNRD1	thioredoxin reductase 1	cytoplasm; mitochondrion; nucleus	cell proliferation; cell redox homeostasis; gastrulation
TXNRD3	thioredoxin reductase 3	cytoplasm; endoplasmic reticulum; nucleus; microsome	cell differentiation; cell redox homeostasis; electron transport chain
UBTF	upstream binding transcription factor, RNA polymerase I	nucleus	regulation of transcription; chromatin silencing at rDNA;
UCHL1	ubiquitin carboxyl-terminal esterase L1	cytoplasm	protein deubiquitination

UCHL5	ubiquitin carboxyl-terminal hydrolase L5	nucleus; cytoplasm	protein deubiquitination
UGDH	UDP-glucose 6-dehydrogenase	cytosol	oxidation reduction; UDP-glucose metabolic process
UPF1	UPF1 regulator of nonsense transcripts homolog	chromatin; cytoplasm; P-body	DNA replication; DNA repair; cell cycle; histone mRNA catabolic process
UQCRQ	ubiquinol-cytochrome c reductase, complex III subunit VII, 9.5kDa	mitochondrion/electron transport chain	ATP synthesis/electron transport chain
UROD	uroporphyrinogen decarboxylase	cytoplasm	heme biosynthetic process; uroporphyrinogen III metabolic process
USP15	ubiquitin specific peptidase 15	not well defined	ubiquitin-dependent protein catabolic process; protein deubiquitination
USP4	ubiquitin specific peptidase 4 (proto-oncogene)	cytoplasm; nucleus	negative regulation of protein ubiquitination; regulation of protein stability
VAMP8	vesicle-associated membrane protein 8	membrane; mitochondrion	vesicle-mediated transport; Cellular Membrane Organization
VARS	valyl-tRNA synthetase	cytoplasm; mitochondrion	valyl-tRNA aminoacylation; translational elongation
VAT1	vesicle amine transport protein 1 homolog	cytoplasm	oxidation reduction
VCAM1	vascular cell adhesion molecule 1	cell membrane; plasma membrane; extracellular space	response to nutrient; response to hypoxia
VCAN	versican	extracellular space/matrix	angiogenesis; cell adhesion; cell proliferation; cell migration/cell-cell migration/collagen
VCL	viniculin	cell junction; cytoplasm; membrane; actin	cell adhesion
VCP	valosin-containing protein	cytoplasm; nucleus	protein ubiquitination
VIM	vimentin	axon; cytoskeletin; colocalizes with plasma membrane; cytoplasm	intermediate filament-based process
VPS25	vacuolar protein sorting 25 homolog	cytoplasm; endocytic vesicle/endosome; membrane; nucleus;	transcription; transport
VPS35	vacuolar protein sorting 35 homolog	cytoplasm; membrane	protein transposrt; retrograde transport, endosome to Golgi

VPS45	vacuolar protein sorting 45 homolog	golgi apparatus membrane; peripheral membrane protein	vesicle docking involved in exocytosis; vesicle medited transport
VTI1A	vesicle transport through interaction with t-SNAREs homolog 1A	golgi apparatus membrane; Single-pass type IV membrane protein. Mainly associated with the Golgi apparatus	vesicle-mediated transport; protein transport; retrograde transport, endosome to Golgi
XDH	xanthine dehydrogenase	peroxisome; cytoplasm	lactation; oxidation reduction; regulation of epithelial cell differentiation; xanthine catabolic process
XPO5	exportin-5	cytoplasm; nucleus	gene silencing by RNA; protein transport
XRCC1	X-ray repair complementing defective repair in Chinese hamster cells 1	nucleus	response to hypoxia; single strand break repair
YAP1	yes-associated protein 1	nucleus; cytoplasm	cell proliferation; hippo signaling cascade; regulation of transcription
YARS	yrosyl-tRNA synthetase	cytoplasm; nucleus	tyrosyl-tRNA aminoacylation; apoptosis
ZFHX3	zinc finger homeobox 3	mitochondrion; nucleus	positive or negative regulation of myoblast differentiation
ZFR	zinc finger RNA binding protein	chromosome;cytoplasm; nucleoplamsm	DNA binding; poly(a) RNA binding protein binding; zinc ion binding
ZMAT3	zinc finger matrin type 3	nuclear matrix, nucleolus	cell growth (negative); protein transport; response to DNA damage
