# Supplementary figures and images for: Identification of AMP-activated protein kinase targets by a consensus sequence search of the proteome
Source: BMC Syst Biol. 2015 Mar 11;9:13. doi: 10.1186/s12918-015-0156-0 (PMC4357066; doi:10.1186/s12918-015-0156-0)

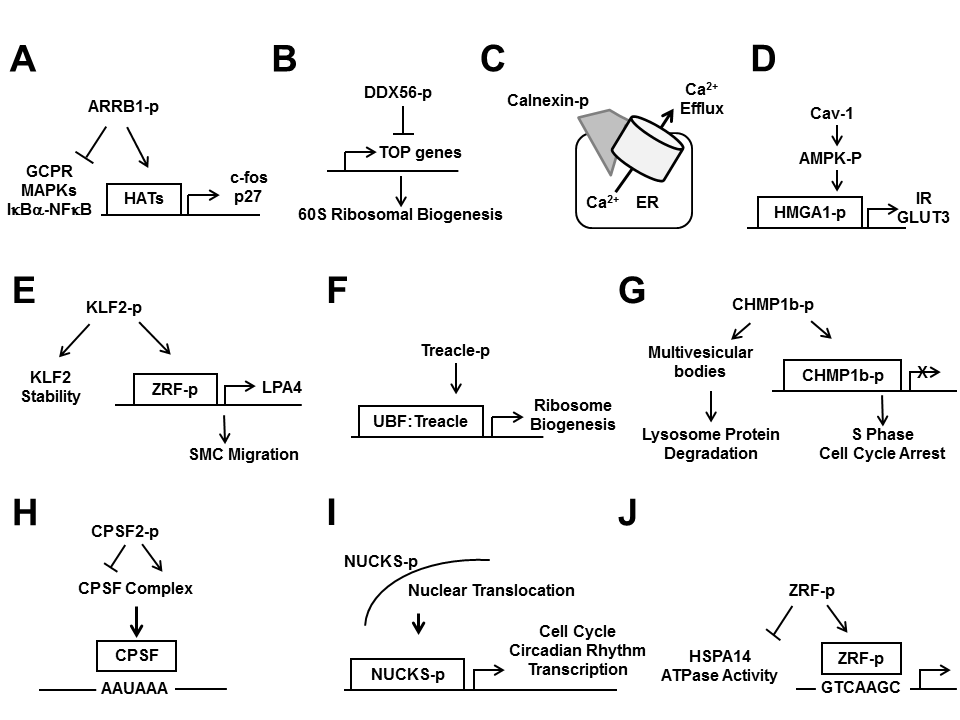

Supplement: Additional file 2: Figure S1. — Predicted modules of AMPK phosphorylated substrates and the related functions. (A) AMPK phosphorylation of ARRB1 inhibits GPCR signaling and increases histone acetylation activity at c-Fos and p27 promoters. (B) AMPK phosphorylation of DDX56 TOP gene translation and subsequent 60S ribosomal biogenesis. (C) AMPK phosphorylation of Calnexin acts a chaperone to positively regulate ER Ca2+ efflux through SERCA2b. (D) AMPK phosphorylation of HMGA1 increases INSR and Glut3 transcription through the Cav-1/AMPK pathway. (E) AMPK phosphorylation of KLF2 increases LP4R transcription and SMC recruitment. (F) AMPK phosphorylation of Treacle associates with UBF to increase AMPK-related gene transcription. (G) AMPK phosphorylation of CHMP1b facilitates lysosomal recycling of proteins and increases S1 cell cycle arrest. (H) AMPK phosphorylation of CPSF2 increases translation of AMPK related genes. (I) AMPK phosphorylation of NUCKS transcriptionally regulates the circadian rhythm. (J) AMPK phosphorylation of ZRF1 inhibits ATPase activity but increases transcription of AMPK-related genes. [file 12918_2015_156_MOESM2_ESM.tiff]
